# Benthic diatom communities and a comparative seasonal-based ecological quality assessment of a transboundary river in Bangladesh

**DOI:** 10.1371/journal.pone.0291751

**Published:** 2023-10-04

**Authors:** Md Mehedi Hasan, Md Ataul Gani, Md Almujaddade Alfasane, Mst Ayesha, Khurshid Nahar

**Affiliations:** 1 Department of Botany, Jagannath University, Dhaka, Bangladesh; 2 Department of Botany, University of Dhaka, Dhaka, Bangladesh; Soil and Water Resources Institute ELGO-DIMITRA, GREECE

## Abstract

Seasonality can play a crucial role in altering water quality in tropical rivers, and as a benthic community, diatom can show seasonal variation and changes in ecological status. During the present study, the Trophic Diatom Index (TDI) and Water Quality Index (WQI) were used to determine the ecological status of a transboundary river, the Sari-Goyain River in Bangladesh. Samplings were carried out from upstream to downstream river sites in wet and dry seasons to observe the seasonal dynamics. The benthic diatom composition and physicochemical parameters showed seasonal variation in ecological water quality assessment. In the River, 42 different diatom species from 19 genera were recorded. The mean TDI values indicated an oligotrophic condition of the river in both seasons. But, the WQI values showed excellent and good water quality in the wet and dry seasons, respectively. So, the WQI was helpful in assessing seasonal variation of ecological water quality status in the Sari-Goyain River. For the long-term monitoring of the ecological status of the river, seasonal variation and WQI-based assessment should be considered.

## Introduction

River ecology can vary due to seasonal changes. This seasonality plays an important role in the quantitative and qualitative assessment of physicochemical parameters and aquatic organisms [1]. Seasonality also has an essential role in altering river flow in tropical rivers, thus restructuring channel morphology, which can impact water quality [2, 3]. In addition, changes in channel morphology can affect the interaction of the benthic community. So, seasonal variation can induce the altered ecological health of the river, showing changes in water quality [4, 5].

Water quality and flow alteration in transboundary river ecosystems are important issues due to water allocation policy, construction of dams and changes in anthropogenic activities. Moreover, the target (6.5) of the sustainable development goal (SDG) on ‘integrated water resource management’ demands the successful allocation of transboundary waters coupled with the health, food and environment [6]. Transboundary rivers of South Asia are facing various complicated challenges, including ecosystem deterioration and water pollution [7]. In that region, Bangladesh has enormous freshwater resources, including 54 transboundary rivers [8]. In the country, these rivers are situated at the downstream part of the Ganges- Brahmaputra- Meghna (BGM) rivers and contribute about 80 per cent of the annual freshwater inflow into the country [9]. The monsoonal rainfall and seasonal flooding affect the river flow systems, leading to changes in the environment and organisms. The life and living of human beings are inherently dependent on these river systems [10]. For that reason, diagnosis of the ecological status of the rivers is crucial for the survival of human beings. Evaluation of ecological status depends on various factors such as water chemistry, physical habitat, climatic and hydrological conditions and biotic composition [5, 11]. Organisms such as diatom, a thoroughgoing benthos component sensitive to water quality changes, influence the variation in river ecological conditions. Factors like temperature, nutrient availability and a few conditions resulting from urbanisation and land-use characteristics are responsible for diatom diversity [12]. Assiduously, seasonal dynamics of diatom and different water parameters are subject to variation in ecological status assessments [13].

Benthic diatom is a good indicator for evaluating environmental conditions [14, 15]. Diatom species are an alarming agent in the aquatic ecosystem for seasonal dynamics due to their different lifestyle, habitat variance and regeneration systems [16]. Diatoms are considered aquatic producer groups that quickly react to changes in environmental variables. Planktonic and benthic diatoms are responsible for seasonal variation in species richness and community compositions, where benthic diatom assemblages become more affected by environmental factors. Because of its strong correlation with ecological factors, benthic diatom can be recommended as a biotic indicator to monitor and assess the ecological condition of the rivers [17, 18]. Microscopic measurement of bio-volume allows high taxonomic resolution up to the species level errors, and biovolume calculation is needed to assess the relative abundance (as biomass or carbon) of benthic diatom (varying in size and shape) [19]. Biomass estimation (biovolume calculation as biomass) is essential for most microbial plankton ecology studies [20].

Ecological quality is forced by seasonality and human activities that may result in biological, physical and chemical pollution, and the resulting parameters can be used to assess water quality. Using benthic diatom, the Trophic Diatom Index (TDI) was developed to evaluate nutrient conditions in freshwater and determine taxonomic changes, which are deeply related to trophic status and regulate the biomass of water bodies [21]. There are some limitations of TDI. For instance, it can not account for changes to organic pollution and resources like habitat loss [22, 23]. Despite the limitations, TDI nowadays has been used in tropical regions to assess water quality. Ecological water quality also demands the use of physicochemical factors of the water body. Based on the physicochemical parameters Water Quality Index (WQI) has been used widely [24, 25] and is considered an effective water quality assessment method to develop the overall status of the water system [26, 27] and can provide information to citizens and policymakers [28]. WQI uses mainly physicochemical parameters for assessing water quality by using a single number for a specific location and time [29]. The physicochemical parameters in water bodies vary in composition and concentration on a seasonal, diurnal basis, thus utilising WQI [5]. These variations may be related to water use and rainfall patterns, resulting in variations in WQI [30].

River resources contribute significantly to the national domestic product [31]. Still, rivers in the country are being polluted continuously with contaminants like organic and inorganic pollutants, mainly untreated industrial effluents, improper disposal of domestic waste and agricultural runoffs. Thus, monitoring water quality is essential for the proper management of river ecosystems in the country [32]. Although different indices and quality elements have been introduced for the ecological quality assessments [33–35], more is needed to conclude which one might be suitable for water quality assessments due to anthropogenic loads. The Sari-Goyain River has excellent recreational value with extensive human interference. The present research is an approach to studying the ecological condition of the Sari-Goyain River in Bangladesh, considering seasonal dynamics of benthic diatom communities and physicochemical factors. To achieve this, a comparative assessment based on TDI and WQI is highlighted in the present research.

## Materials and methods

### Study area

**Fig 1:**
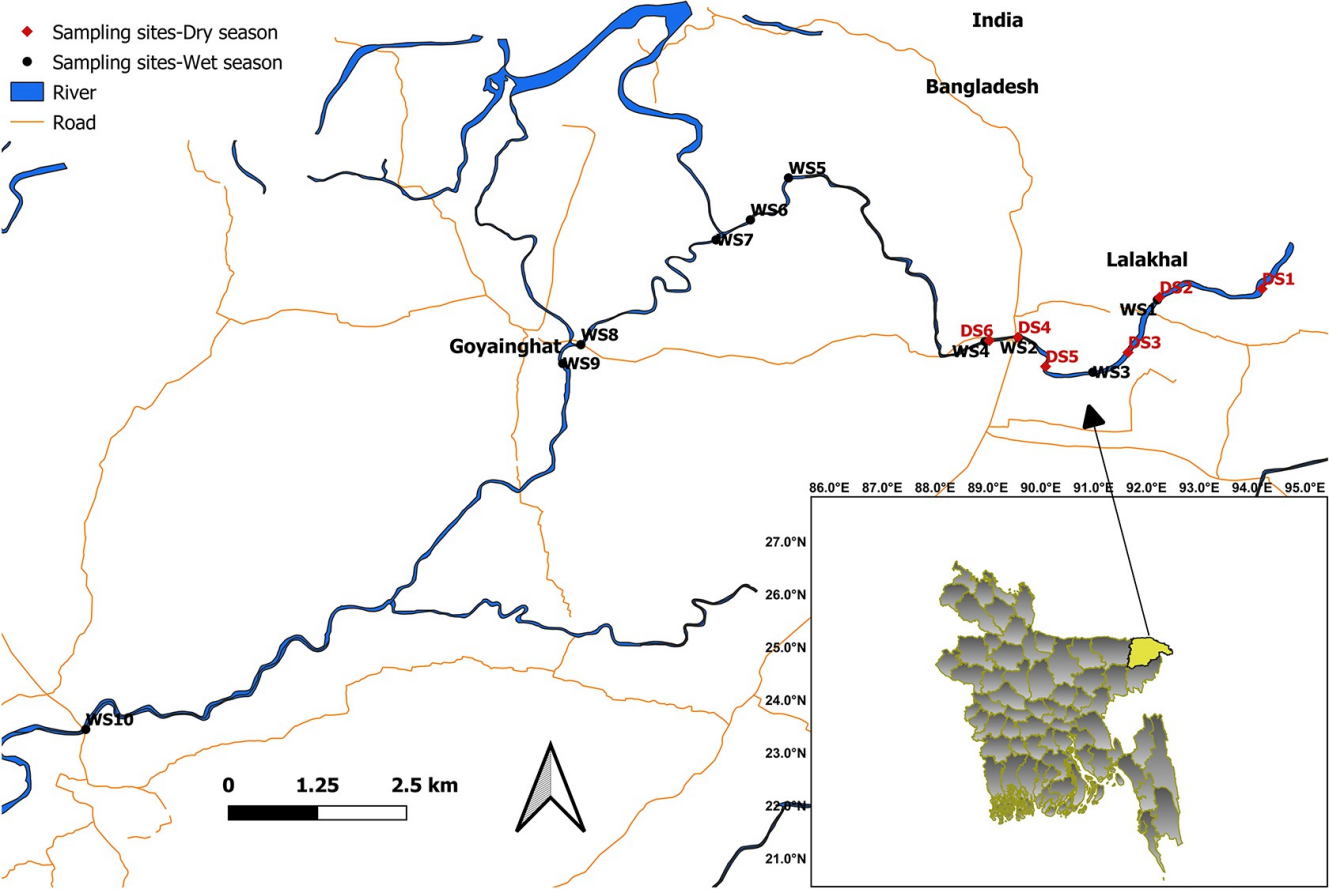
Sampling locations in Sari-Goyain River, Sylhet, Bangladesh (Source: GeoDASH, https://geodash.gov.bd).

The Sari-GoyainRiver is about 45 km from Sylhet city, adjacent to the Tamabil road. Different places of the Sari-Goyain River with enormous ecological variation were selected for the investigation, and sampling sites were recognised using Google Earth. Based on ecological variations, ten sites (WS1, WS2, WS3, WS4, WS5, WS6, WS7, WS8, WS9 WS10) during the wet season (May 2017) and six sites (DS1, DS2, DS3, DS4, DS5 and DS6) during the dry season (February 2018) were selected (Fig 1). All the sampling sites were sedimented with silt and clay except WS1, WS2, WS5, DS2, DS3 and DS4, mainly sand. Riparian tree cover was present except for the downstream sites of WS8, WS9 and WS10. Tree roots, leaf litter and woody branches were found in most sampling sites. The river bed of sampling sites WS1 to WS3 and DS1 to DS5 was shaded with some open areas, while the rest of the sites consisted of large open areas.

### Field samplings

Samples of water and sediment (mud) were collected during the wet season (May 2017) and dry season (February 2018) to assess the various ecological characteristics of the Sari-Goyain River in Sylhet, Bangladesh, and field samplings were carried out accordingly. *In situ* measurements of air and water temperature (alcoholic thermometer), pH (HI-96107, Hanna), conductivity and total dissolved solids (TDS, EC 300-YSI, USA), and dissolved oxygen (DO, DO200A-YSI, USA) were measured using portable devices.

Water samples were directly collected from the one-meter depth of the river for physicochemical analysis. Surface sediment (mud) was collected from the benthic zone by a large spoon (length approx. 50 cm, diameter approx. 5 mm) poured into a plastic bottle underwater for the benthic diatom quantification. The bottle was covered by a cap. A few drops of formaldehyde were added to the mud sample and carried to the laboratory for diatom analysis.

### Lab analysis

Whatman GF/C 4.7 cm circles were used in the laboratory to filtrate the water sample. The concentration of nitrate-nitrogen (NO_3_-N) in the filtered sample water was determined following [36]. Soluble reactive phosphorus (SRP) determination was followed after [37]. The determination of soluble reactive silicate (SRS) was followed after [38].

Clean frustules of diatoms were prepared to treat the sediment cores by the wet combustion method [39, 40]. First, 1 g of sediment sample was taken in a Pyrex test tube, and then 30% hydrogen peroxide and potassium dichromate was added. After combustion, 30 ml of distilled water was added and kept at room temperature for a day. The next day, water above the sediment was poured down and done three times. After cleaning, the well-mixed suspension was taken on HBCC and covered with a cover glass to count diatom.

#### Diatom counting and biovolume calculation

Counting of diatom was done with the help of an HBCC (Helber Bacteria Counting Chamber, Z30000, Hawksley, UK). HBCC has a grid of known volumes by which the number of diatoms per μL can be calculated, and double ruled lines marked off this chamber into nine large squares in three-by-three patterns. Each large square contains 16 small squares, making 144 small squares ((9*16) under a compound microscope. The microscope magnification was 400X and fitted with Japan’s Nikon FX-35WA camera. The cell density of diatoms was calculated as density as ind/g sediment (obtained from ind/ml) = (v/0.0216 μL × total count), where v = mud/sediment aliquot volume inμl. 0.0216 μl is the volume of the counting chamber for three replicate counting (volume of 9 large squares = 00.0072 μL). Total count = total number of individuals present in three replicate counting and expressed as ind/L (S1 Table). Diatoms were identified according to [41, 42]. The bio-volume of the diatom was calculated using appropriate geometric formulae [19]. The length, breadth or diameter was measured under high magnification of a compound microscope. The height of the diatom species was considered from the existing sources for bio-volume calculation [43] and expressed as μm^3^/L (S2 Table).

#### Dominance index

The dominance index was applied to identify dominant diatom species in different seasons, using the following formula [44]-

$$Dominance\;index=\left(\frac{ni}{N}\right)\times fi$$

Where ni = the total number of cells in species i, N = the total number of cells in all species, and fi = frequency of occurrence of species i.

### Water quality indices

Water Quality Indices are considered an effective method to measure water quality. The indices are included with a mathematical equation to determine water quality and conclusions about water status. In the Sari-Goyain River, the Trophic Diatom Index (TDI) and Water Quality Index (WQI) were applied to assess the ecological status.

#### TDI (Trophic Diatom Index)

Trophic Diatom Index (TDI) was developed for European rivers but nowadays has extensively been used for tropical streams such as the Indian sub-continent and sub-tropical Australia [45–49]. TDI monitors the trophic status of rivers and water quality based on diatom composition and pollution sensitivity, ranging from 1 to 5 [50, 51]. The limit for ecological and trophic status is shown in Table 1.

TDI=(WMS*25)−25WMS=∑AiViSi/∑AiVi

Where, *A*_*i*_ = abundance (proportion) of species in the sample, *V*_*i*_ = indicator value and *S*_*i*_ = pollution sensitivity of species i.

**Table 1:** Class limit value ecological and trophic status for diatom index (TDI) according to [42, 43].

TDI score (0–100)	Water quality class	Ecological status	Trophic status
< 35	i. class	High	Oligotrophic
35–50	ii. class	Good	Oligo-Mesotrophic
50–60	iii. class	Moderate	Mesotrophic
60–75	iv. class	Low/Poor	Eutrophic
>75	v. class	Bad	Hypertrophic

#### Water quality index (WQI)

The Water Quality Index (WQI) calculation needs physicochemical parameters representing water quality [52]. Different physicochemical parameters such as pH, DO, TDS, conductivity, SRS, SRP and NO_3_-N of water samples were used to calculate the WQI value at each site. According to the degree of purity, the WQI method classifies the water quality [Table 2, 53].

WQI=∑QiWi/∑Wi

*Q*_*i*_ = The quality rating scale for each parameter is calculated by Q_i_ = 100[(V_i_-V_o_/S_i_-V_o_)]

*V*_*i*_ is the estimated concentration of the ith parameter in the analysed water; *V*_*o*_ is the ideal value of this parameter in pure water; *V*_*o*_ = 0 (except pH = 7.0 and DO = 14.6 mg/l); *Si* recommended standard value of the ith parameter.

**Table 2:** Status of water quality limit value and status based on the arithmetic WQI method according to [53].

Water quality index value	Status
0–25	Excellent
26–50	Good
51–75	Poor
76–100	Very poor
> 100	Unsuitable for drinking and propagation of fish culture

### Statistical analysis

All statistical analysis was performed in R v4.1.2 [54]. The R function "prcomp" was used for the PCA analysis, and the R function"ggbiplot", which is available on GitHub ("vqv/ggbiplot"), was used to visualise the PCA plot. Based on the eigen vectors of the variables in the first two PC axes, factors were selected and rerun the PCA, which showed a better result than the preliminary one. Pearson correlation was performed among the selected physicochemical variables based on PCA, abundance and biovolume of benthic diatom, WQI and TDI. The R function “ggcorrmat” in the package "ggstatsplot" was used to display a correlation matrix chart with adjusted p-values by Holm’s method. Before analysis, all the parameters were transformed into a log (x+1) except pH, air and water temperatures, which were standardised.

## Results

### Benthic diatom composition and their seasonal dynamics

**Fig 2:**
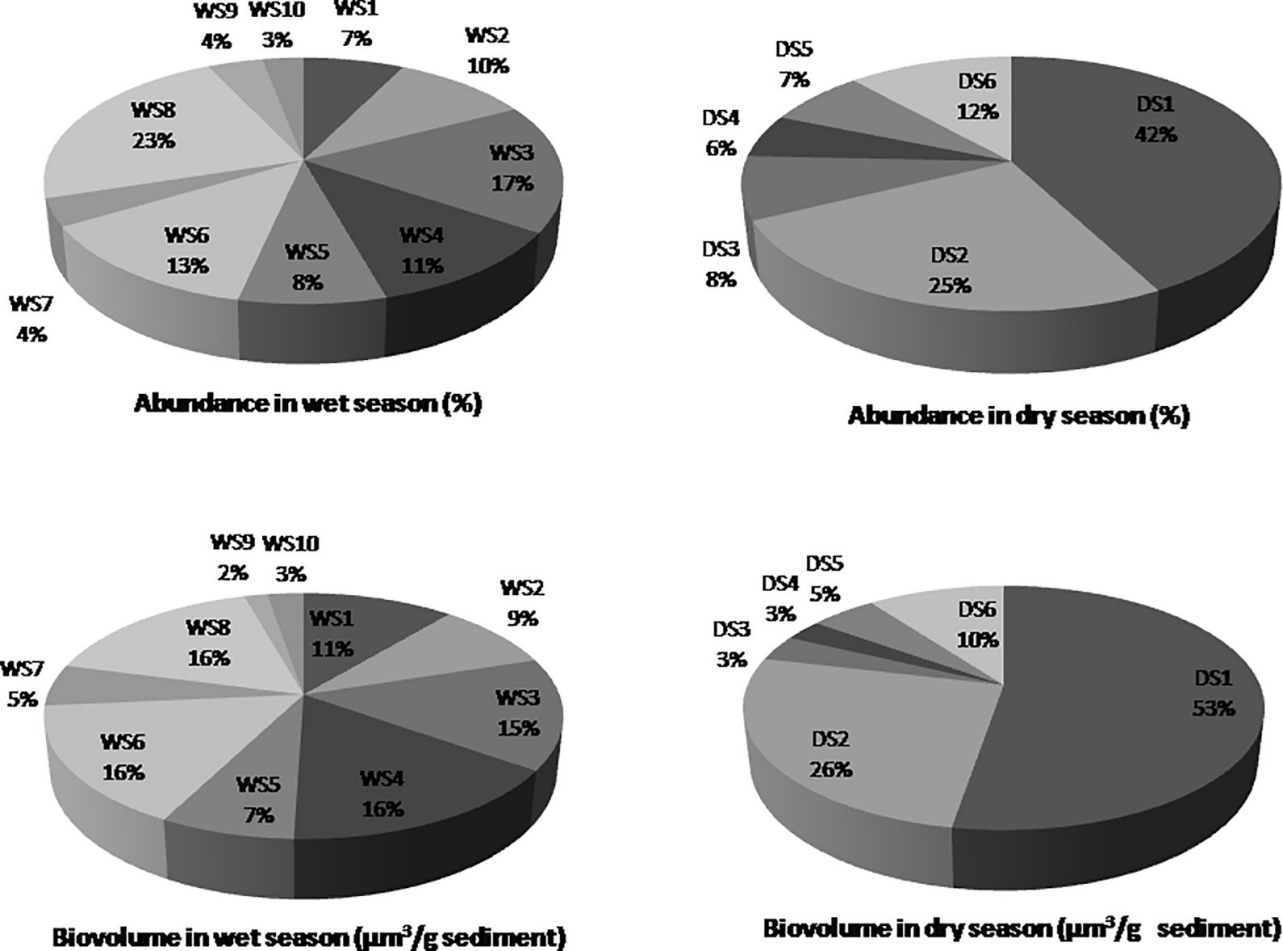
Abundance and biovolume of benthic diatom in different sites of Sari-Goyain River during wet and dry seasons.

The benthic diatom diversity in the Sari-Goyain River was represented by 42 species belonging to 19 genera, of which 13 genera were common for both wet and dry seasons. Among 42 species, 19 were common for both seasons; 11 were only found in the wet season and 12 in the dry season. During the wet season, the highest percentage of diatom abundance was found in WS8 (23%), followed by WS3 (17%), WS6 (13%), WS4 (11%), WS2 (10%), WS5 (8%), WS1(7%), WS9 (4%), WS7 (4%) and WS10 (3%). The diatom biovolume did not show the same hierarchy as abundance; instead, the highest biovolume was 16% and was observed in WS4, WS6 and WS8. However, the lowest biovolume was found to be like abundance in WS10 (3%). During the dry season, the highest abundance was observed in DS1 (42%), followed by DS2 (25%), DS6 (12%), DS3 (8%), DS5 (7%) and DS4 (3%). In the dry season, the biovolume showed almost the same distribution, where the highest number was in station DS1 (53%), followed by DS2 (26%), DS6 (10%), DS5 (5%) and the lowest in DS3 and DS4 (3%) (Fig 2). The complete list of benthic diatom with abundance and biovolume was provided in S1 and S2 Tables.

The most abundant and dominant diatom species in both wet and dry seasons was *Fragilaria capucina var*. *vaucheriae*. However, the abundance, dominance index, and bio-volume were higher in the dry season. *Encyonema elginense*, *Synedra ulna var*. *contracta and Gomphonema insigne* were the dominant species during the wet season, and *Eunotia minor*, *Sellaphora americana* and *Iconella splendida* were the dominant species during the dry season according to the dominance index. Among the dominant species, *Fragilaria capucina var*. *vaucheriae* was common in both seasons, and the abundance was higher in the dry season (2.08 ×10^3^ ind/g sediment) than in the wet season (0.34×10^3^ ind/g sediment) (Table 3).

**Table 3:** Abundance (×10^3^ ind/g sediment) and biovolume (μm^3^/g sediment) of dominant benthic diatom in Sari-Goyain River during wet and dry seasons with dominance index.

Species	Dominance index		Abundance		Biovolume	
	Wet	Dry	Wet	Dry	Wet	Dry
*Encyonema elginense* (Krammer) D. G. Mann	0.12	-	0.32	-	636.19	-
*Eunotia minor* (Kutzing) Grunow	-	0.11	-	0.62	-	251.45
Fragilaria capucina var. vaucheriae (Kützing) Lange-Bertalot	0.13	0.17	0.34	2.08	1862.04	11395.31
*Gomphonema insigne* W. Gregory	0.10	-	0.23	-	406.68	-
*Sellaphora americana* (Ehrenberg) D. G. Mann	-	0.11	-	0.44	-	2467.24
*Iconella splendida* (Ehrenberg) Ruck & Nakov	-	0.16	-	0.63	-	581.99
*Synedra ulna var*. *contracta* Ostrup	0.10	-	0.23	-	304.98	-

### Seasonal dynamics of physicochemical factors

**Fig 3:**
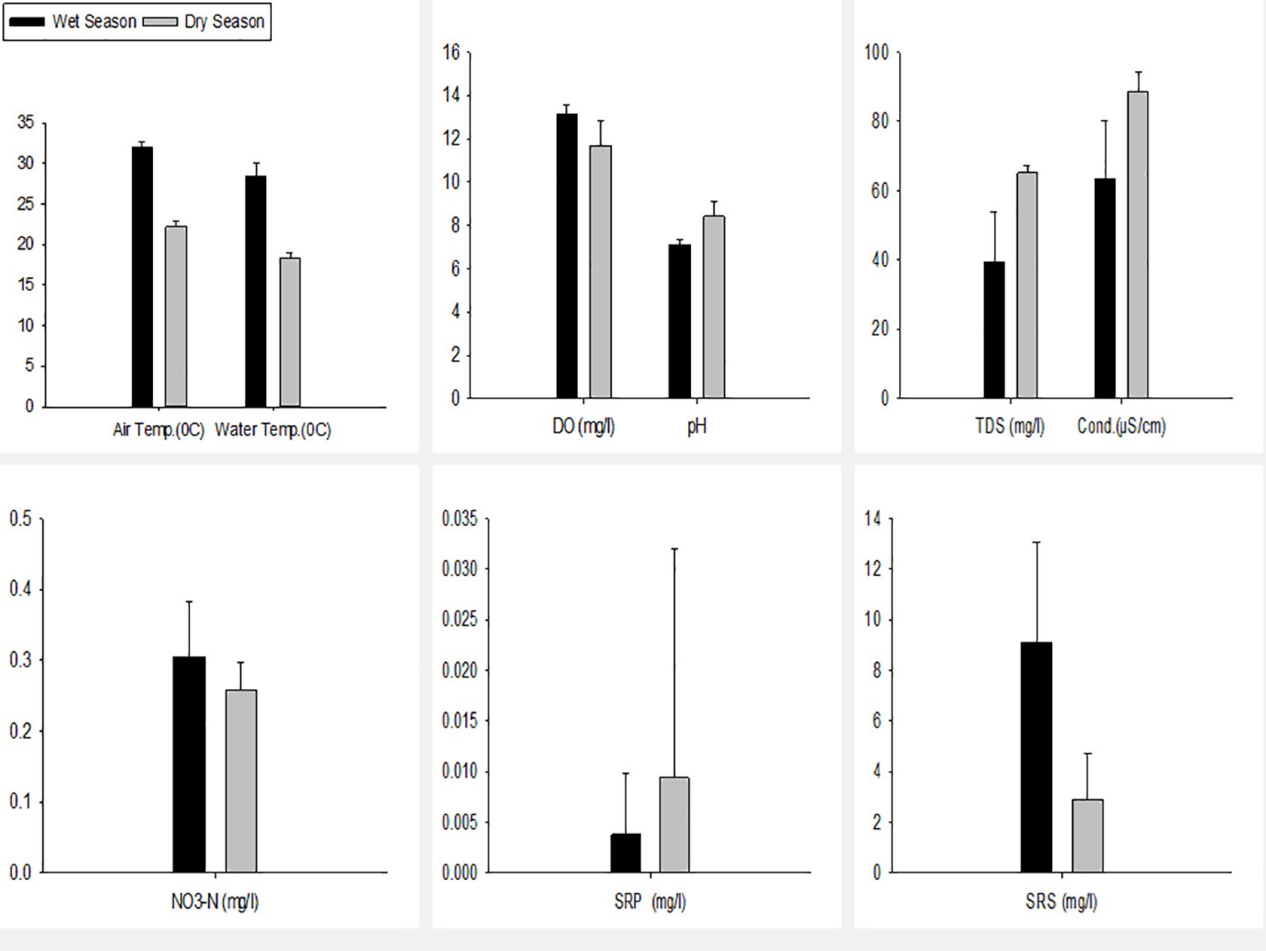
Different physicochemical parameters of the Sari-Goyain River during wet and dry seasons.

The present study exhibits seasonal variation among different physicochemical parameters. Air temperature, water temperature, pH, TDS, conductivity, DO, SRS, SRP and NO_3_-N showed a notable seasonal variation. The average air temperature was higher in the wet season (31.98 ⁰C) than in the dry season (22.23 ⁰C). The highest air temperature during the wet season in WS4 (33.1 ⁰C) and the lowest in WS1(30.9⁰C). The highest water temperature in the dry season was found in DS2 (23.5 ⁰C) and the lowest in DS6 (21.5 ⁰C). The water temperature showed a similar pattern, higher in the wet season (28.4 ⁰C) than in the dry season (18.38 ⁰C), but the spatial variation differed. The maximum temperature was recorded in WS10 (30.7 ⁰C) and DS5(19.2 ⁰C), and the minimum in WS1 (25.1 ⁰C) and DS1 (17.7 ⁰C) during the wet and dry seasons, respectively. DO, SRS and NO_3_-N showed higher average values (13.14mg/l; 0.31 mg/l) in the wet season than in the dry season (11.7mg/l; 0.26 mg/l). In the wet season, the highest NO_3_-N concentration was measured in WS1 and WS2 (0.43mg/l) and the lowest in WS3, WS9 and WS10 (0.23 mg/l). The highest concentration was observed in DS4 (0.30 mg/l), and the lowest was in DS1 (0.21 mg/l) during the dry season. pH, TDS, conductivity and SRP values were higher in the dry season than in the wet season (Fig 3).

### Seasonal variation of TDI

TDI is used widely to monitor trophic status based on diatom composition. The identified pollution-tolerant taxa in the river were *Gomphonema insigne*, *Sellaphora pupula*, *Craticula cuspidata*, *Sellaphora americana* and *Nitzschia sociabilis*. According to the TDI, all the sites assessed as oligotrophic except WS2 were assessed as oligo-mesotrophic during the wet season. Other sites showed oligotrophic status during the dry season except for DS4 and DS6. So, the overall ecological quality of Sari- Goyain was assessed as oligotrophic in both seasons (Table 4).

**Table 4:** TDI value, water quality class, ecological status and trophic status of Sari-Goyain River in Bangladesh during wet and dry seasons.

	Wet Season					Dry Season			
Station	TDI Score	Water Quality Class	Ecological Status	Trophic Status	Station	TDI Score	Water Quality Class	Ecological Status	Trophic Status
WS1	25	Class I	High	Oligotrophic	DS1	21.94	Class I	High	Oligotrophic
WS2	36.67	Class II	Good	Oligo-Mesotrophic	DS2	25.56	Class I	High	Oligotrophic
WS3	15.91	Class I	High	Oligotrophic	DS3	31.25	Class I	High	Oligotrophic
WS4	22.06	Class I	High	Oligotrophic	DS4	37.5	Class II	Good	Oligo-Mesotrophic
WS5	26.79	Class I	High	Oligotrophic	DS5	32.35	Class I	High	Oligotrophic
WS6	23.75	Class I	High	Oligotrophic	DS6	42.22	Class II	Good	Oligo-Mesotrophic
WS7	32.69	Class I	High	Oligotrophic		-	-	-	-
WS8	20.41	Class I	High	Oligotrophic		-	-	-	-
WS9	19.44	Class I	High	Oligotrophic		-	-	-	-
WS10	18.75	Class I	High	Oligotrophic		-	-	-	-
Overall	24.15	Class I	High	Oligotrophic		31.8	Class I	High	Oligotrophic

### Seasonal variation of WQI

The Water Quality Index (WQI) represents the water status of different sampling sites of the Sari-Goyain River. In the wet season, all the sites exhibited excellent ecological status ranging the WQI value from 11.23 (WS9) to 24.68 (WS4). Only two sampling sites showed excellent status (DS1 and DS2). In contrast, others showed good status. Overall, the WQI index indicated excellent water quality during the wet season and good water quality during the dry season (Table 5).

**Table 5:** Status of water quality of Sari-Goyain River in Bangladesh during wet and dry seasons based on WQI.

Wet Season			Dry Season		
Station	WQI value	Water quality status	Station	WQI value	Water quality status
WS1	7.84	Excellent	DS1	16.99	Excellent
WS2	9.96	Excellent	DS2	8.57	Excellent
WS3	11.72	Excellent	DS3	40.44	Good
WS4	18.14	Excellent	DS4	38.75	Good
WS5	6.26	Excellent	DS5	40.05	Good
WS6	4.4	Excellent	DS6	40.09	Good
WS7	17.6	Excellent	-	-	-
WS8	11.88	Excellent	-	-	-
WS9	3.93	Excellent	-	-	-
WS10	7.9	Excellent	-	-	-
Overall	9.96	Excellent		30.81	Good

### Correlation of different physicochemical parameters with benthic diatom, TDI and WQI

**Fig 4:**
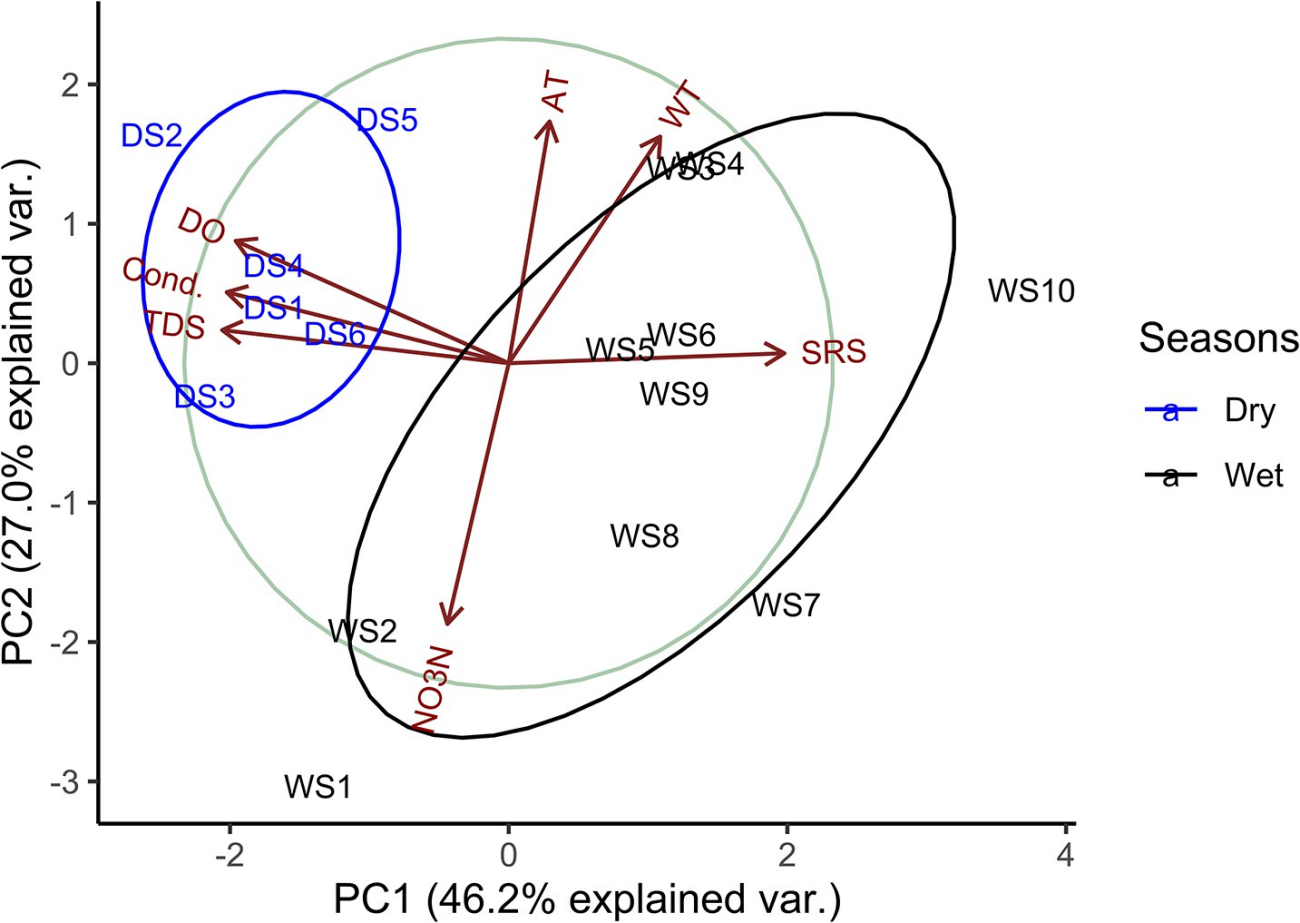
PCA ordination plot of sampling sites with selected physicochemical factors. AT = air temperature, WT = water temperature and Cond. = conductivity.

In PCA, the first two axes explained 73.15% of the variance, and the eigenvalue of the PC1 and PC2 was 1.79 and 1.37, respectively. Air (r = 0.74) and water (r = 0.69) temperatures correlated positively with PC2, while NO_3_-N (r = -0.80) correlated negatively. TDS (r = -0.88), conductivity (r = -0.87), and DO (r = -0.84) correlated negatively with PC1, and SRS (r = 0.85) correlated positively. In the PCA plot, wet and dry sampling sites were grouped separately and showed correlations with different physicochemical factors. The dry season’s sampling sites were mainly influenced by DO, TDS and conductivity, whereas the wet season’s sampling sites were mainly by water temperature, NO_3_-N and SRS concentration (Fig 4).

**Fig 5:**
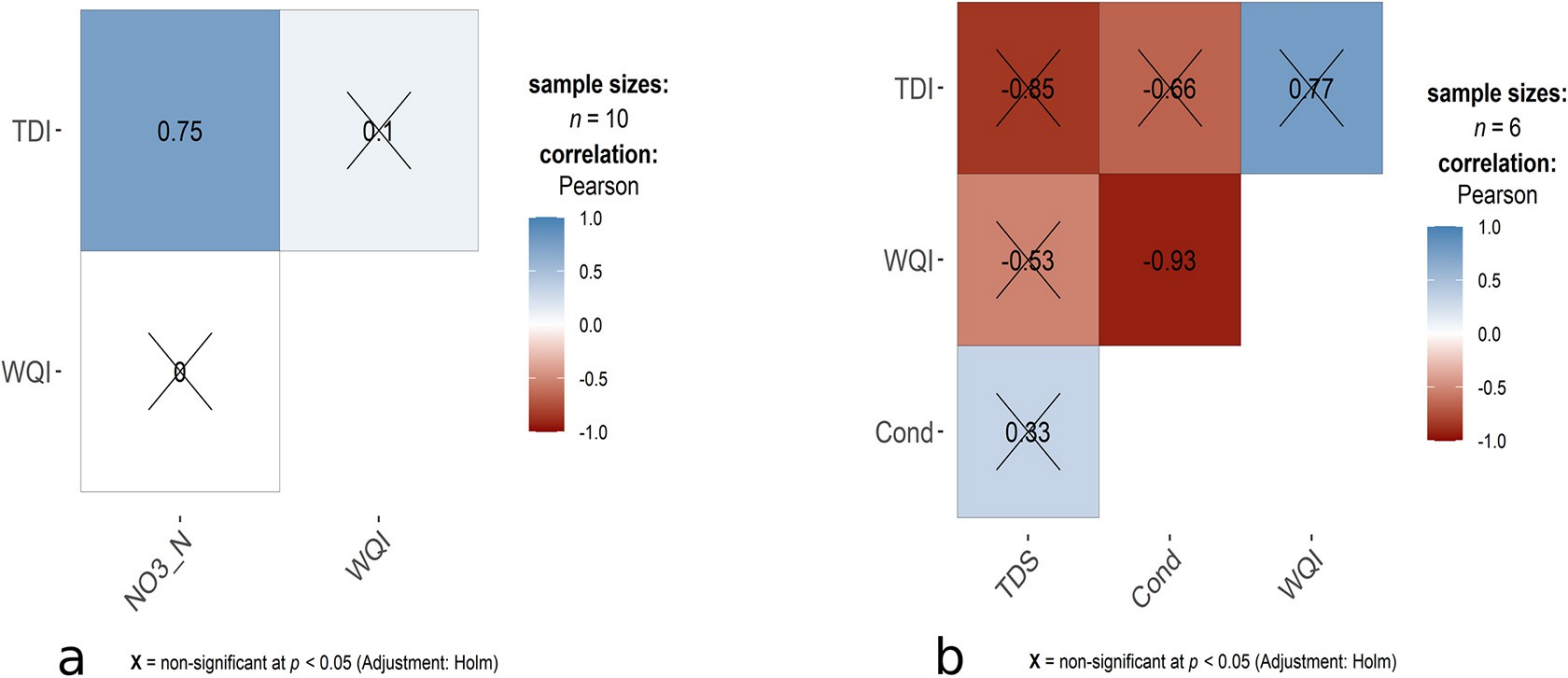
Pearson correlation between selected physicochemical variables, WQI and TDI during wet (a) and dry (b) seasons.

Pearson correlation showed different results in wet and dry seasons except for the correlation between abundance and biovolume. During the wet season, NO_3_-N (r = 0.75) positively correlated with TDI, and in the dry season, conductivity (r = -0.93) negatively correlated with WQI (Fig 5).

## Discussion

Diatoms play a significant role as an environmental indicator due to their exhibition of seasonal variation, although their density was lower in riverine systems than in lentic systems [55, 56]. The average abundance of benthic diatom in the Sari-Goyain River varied from wet to dry seasons (Fig 2, Table 3). Different studies showed that changes in abundance and biovolume of benthic diatom assemblages occurred due to seasonal variation representing the dominance of one species over another [57–59]. During the present investigation, *Fragilaria capucina var*. *vaucheriae* was the most abundant species of both seasons, usually epilithic, found in calcareous springs and streams. This diatom prefers to live in eutrophic waters [60]; due to this, the abundance was higher in the dry season (Table 3). *Encyonema elginense* was another dominant species in the wet season, a crucial constituent of the food web in water bodies and acts as an indicator for changes in water temperature and nutrient levels [61]. Therefore, this diatom was only found in the wet season, indicating a good predictor for seasonal variation in the Sari-Goyain River. *Eunotia minor* mainly occurs at low pH, but during the present study, it was recorded in the dry season when pH was comparatively higher [62]. Other dominant diatom species, *Gomphonema insigne*, *Sellaphora americana*, *Iconella splendida*, and *Synedra ulna var*. *contracta*, seem to grow well in response to nutrients. Several studies showed that these freshwater diatoms act as bio-indicators for pollution changes [61, 63].

TDI is a powerful tool for monitoring eutrophication and water quality and recognising disturbance systems in tropical regions [45, 64]. A case study in Al-Sabil River showed that TDI values varied from 23.33 to 55.54. The trophic status was oligotrophic to mesotrophic with free or low pollution, suitable for living well and consisted of few nutrients. According to the TDI value in the Al-Sabil River in Iraq, the water quality level ranging from oligotrophic to mesotrophic means water was quality medium to good [65]. Moreover, Diatoms are the primary producer and suitable indicator for exhibiting seasonal variation, which can influence the ecological status of the water body [66].

TDI represented the Sari Goyain River in oligotrophic conditions in both seasons. However, few sampling sites (WS1, DS4 and DS6) showed oligo-mesotrophic status, which indicated the deterioration of the water quality in some areas of the river due to anthropogenic stresses such as anchoring of boats, sewage water pollution, tourist pressure, collection of the ornamental shell etc. During the field study, a highway bridge was observed between the DS4 and DS6, and the riverbank was modified accordingly. In addition, these sites experienced pollution pressure due to touristic activities.

WQI delivered a message about the effects of different eliminates in water and provided information about water status. The research conducted countrywide in 24 randomly selected Upazilas in Bangladesh found the water quality status "excellent" to "poor" according to WQI [67]. The overall WQI value in both seasons indicated excellent and good water quality status during the present study, clearly showing seasonal variation in water quality status. Seasonal variation of WQI was also observed in another transboundary river in Bangladesh [68].

In the Sari-Goyain River, WQI was more sensitive to assessing water quality, although TDI and WQI provided the same ecological status in the wet season. Comparatively lower WQI values in the wet season showed that dilution of physicochemical factors such as pH, TDS, conductivity and SRP occurred in this season, resulting in better water quality than in the dry season. Different studies showed that some physicochemical factors were concentrated during the dry period, deteriorating water quality [69–72]. The PCA ordination plot showed that wet season sampling sites mainly influenced NO_3_-N and SRS (Fig 4), and the mean value of NO_3_-N and SRS was also higher in wet seasons (Fig 3). This occurred due to high water flow increases in the rate of sediment transport which can increase the concentration of NO_3_-N and SRS. TDI was developed based on the abundance of diatom, so an increase in NO_3_-N and SRS concentration in the wet season boosted the development and distribution of benthic diatom and the result of the correlation matrix supported this also. During the dry season, a significant correlation was found between conductivity and WQI, which might be due to the higher value of conductivity (Fig 5). However, WQI can show effective seasonal variation because of the direct alteration of different physicochemical factors that impact the WQI than the abundance of benthic diatoms.

WQI is a widely used index, and application to the aquatic systems in Bangladesh has already been initiated. We compared our results to other co-workers in the relevant research and found that the calculated value was within the range [67, 68]. But to better estimate in future, it can be calibrated with other river systems of the country, and thus, the selection of different physicochemical parameters will be done more accurately, which is the main drawback of WQI. The TDI we used for the first time to assess water quality in the country, and there needs to be more information on the ecology of benthic diatoms. So, it was not possible to compare the results with existing works in the country. But for future river monitoring programs, TDI can be calibrated and implemented accordingly.

## Conclusion

The abundance, bio-volume of benthic diatom, dominance index, and WQI showed a clear sign of seasonal variation in the assessment of the ecological status of the Sari-Goyain River. However, the TDI did not show the seasonal variation exactly. According to TDI, the Sari-Goyain River is in oligotrophic condition in wet and dry seasons, representing class I water quality with high ecological status. However, excellent and good water quality status was observed in the wet and dry seasons, respectively, based on WQI. The present study showed that WQI performed better in assessing water quality due to anthropogenic pollution, where seasonality plays a crucial role. There was a disturbance on the Sari-Goyain River, but the ecology is in good condition. Extensive human interference should be controlled, and WQI-based monitoring should be implemented to maintain the good ecological state of the river and ecosystem services.

## Supporting information

S1 Table The abundance of benthic diatom (x10^3^ ind/L) in the Sari-Goyain River, Bangladesh during the wet and dry seasons. (DOCX)

S2 Table The biovolume of benthic diatom (x10^3^ μm^3^/L) in the Sari-Goyain River, Bangladesh during the wet and dry seasons. (DOCX)

S1 Appendix Calculation of TDI and WQI in the sampling sites of Sari-Goyain River during the wet and dry seasons. (DOCX)
