# Supplementary material for: Benthic diatom communities and a comparative seasonal-based ecological quality assessment of a transboundary river in Bangladesh
Source: PLoS One. 2023 Oct 4;18(10):e0291751. doi: 10.1371/journal.pone.0291751 (PMC10550107; doi:10.1371/journal.pone.0291751)
Supplement: S1 Table — (DOCX) [file pone.0291751.s001.docx]

**Table S1a.The abundance of benthic diatom (x10³ ind/L) in the Sari-Goyain River, Bangladesh during the wet season.**

| Diatom | Sampling sites | | | | | | | | | |
| --- | --- | --- | --- | --- | --- | --- | --- | --- | --- | --- |
|  | WS1 | WS2 | WS3 | WS4 | WS5 | WS6 | WS7 | WS8 | WS9 | WS100 |
| *Amphora libyca* Ehrenberg | 0 | 0 | 0 | 0 | 0 | 0 | 0 | 0.183 | 0 | 0 |
| *Cymbellacursiformis*L.Hufford & Collins | 0.166 | 0 | 0.2 | 0 | 0 | 0 | 0 | 0.183 | 0 | 0 |
| *Encyonematurgidum* (Gregory) Grunow | 0 | 0.25 | 0.6 | 0.65 | 0.433 | 0.433 | 0 | 0.733 | 0 | 0.15 |
| *Diploneis ovalis* (Hilse) Cleve | 0 | 0 | 0 | 0 | 0 | 0 | 0 | 0.183 | 0 | 0 |
| *Eunotiamarina* Schrader | 0 | 0 | 0.2 | 0 | 0 | 0 | 0 | 0 | 0 | 0 |
| *Eunotia minor (*Kutzing) Grunow | 0.333 | 0 | 0.2 | 0 | 0 | 0.216 | 0 | 0 | 0 | 0.15 |
| *Eunotia veneris* (Kutzing) De Toni | 0 | 0 | 0 | 0 | 0 | 0 | 0 | 0.55 | 0 | 0 |
| *Fragilaria capucina var. vaucheriae*(Kützing) Lange-Bertalot | 0.333 | 0 | 0.6 | 1.08 | 0.216 | 0.65 | 0.15 | 0.366 | 0 | 0 |
| *Gomphomematergestinum* (Grunow) M.Schmidt | 0 | 0 | 0.2 | 0 | 0 | 0 | 0 | 0 | 0 | 0 |
| *Gomphonemaangustatum* [(Kütz.) Rabenh.](https://www.itis.gov/servlet/SingleRpt/RefRpt?search_type=author&search_id=author_id&search_id_value=161690) | 0 | 0 | 0 | 0 | 0 | 0 | 0 | 0.55 | 0 | 0.15 |
| *Gomphonemaincognitum* Reichardt, Jüttner & E.J.Cox | 0 | 0 | 0.4 | 0 | 0 | 0 | 0 | 0 | 0 | 0 |
| *Gomphonema insigne* W. Gregory | 0.166 | 0.75 | 0 | 0 | 0 | 0.216 | 0.15 | 0.916 | 0 | 0.15 |
| *Gomphonemalongiceps* (Ehrenberg) | 0 | 0 | 0 | 0 | 0.65 | 0.433 | 0 | 0 | 0 | 0 |
| *Gomphonema olivaceoides* Hustedt | 0 | 0 | 0.2 | 0 | 0 | 0 | 0 | 0 | 0 | 0 |
| *Aulucoseira grunulutu* (Ehrenberg) | 0 | 0 | 0 | 0 | 0.216 | 0 | 0 | 0 | 0 | 0 |
| *Sellaphora Americana* (Ehrenberg) D.G.Mann | 0.5 | 0 | 0 | 0 | 0 | 0.216 | 0 | 0 | 0 | 0 |
| *Craticula cuspidata* (Kutzing) D.G.Mann | 0 | 0.25 | 0.2 | 0.216 | 0 | 0.433 | 0 | 0.366 | 0.2 | 0 |
| *Luticolacohnii* (Hilse) Mann var. cohnii | 0 | 0 | 0 | 0 | 0 | 0 | 0 | 0 | 0 | 0.15 |
| *Neidiumampliatum* (Ehrenberg) Krammer | 0 | 0 | 0 | 0 | 0 | 0 | 0.3 | 0 | 0 | 0 |
| *Pinnularia acrosphaeria* W. Smith | 0 | 0 | 0 | 0 | 0.433 | 0 | 0 | 0 | 0 | 0 |
| *Pinnularia braunii* (Grunow) Cleve | 0 | 0 | 1.2 | 0.216 | 0 | 0 | 0 | 0.55 | 0.4 | 0 |
| *Pinnulariahemiptera* [(Kütz.) Rabenh.](https://www.itis.gov/servlet/SingleRpt/RefRpt?search_type=author&search_id=author_id&search_id_value=161690) | 0 | 0 | 0 | 0.216 | 0 | 0 | 0 | 0 | 0 | 0 |
| *Pinnularia major* (Kützing) Rabenhorst | 0.166 | 0.5 | 0.2 | 0 | 0 | 0.433 | 0 | 0 | 0 | 0 |
| *Pinnularia pulchra* Oestrup | 0 | 0 | 0 | 0 | 0 | 0 | 0.15 | 0 | 0 | 0 |
| *Pinnularia sp* | 0 | 0 | 0 | 0 | 0 | 0 | 0 | 0.183 | 0 | 0 |
| *Stauroneis schroederi* Hustedt | 0 | 0 | 0 | 0 | 0 | 0 | 0.15 | 0 | 0 | 0 |
| *Strauroneis anceps* Ehrenberg | 0 | 0.25 | 0 | 0 | 0 | 0 | 0 | 0 | 0 | 0 |
| *Iconella splendida* (Ehrenberg) Ruck &Nakov | 0 | 0 | 0 | 0 | 0 | 0 | 0 | 0.183 | 0 | 0 |
| *Fragilaria ulna*(Nitzsch) Lange-Bertalot | 0 | 0.5 | 0 | 0.433 | 0 | 0.216 | 0 | 0.733 | 0.4 | 0 |
| *Fragilaria* *capucina* Desmazières | 0.166 | 0 | 0 | 0 | 0 | 0 | 0 | 0 | 0 | 0 |

**Table S1b. The abundance of benthic diatom (x10³ ind/L) in the Sari-Goyain River, Bangladesh during the dry season.**

| Diatom | Sampling sites | | | | | |
| --- | --- | --- | --- | --- | --- | --- |
|  | DS1 | DS2 | DS3 | DS4 | DS5 | DS6 |
| *Amphora libyca* Ehrenberg | 0 | 0.43 | 0 | 0 | 0 | 0 |
| *Cymbella tumida* (Brébisson) Van Heurck | 0 | 0.216 | 0 | 0 | 0 | 0 |
| *Encyonema turgidum* (Gregory) Grunow | 0.6 | 0.866 | 0.433 | 0 | 0.5 | 0 |
| *Diploneis ovalis* (Hilse) Cleve | 0.2 | 0.216 | 0 | 0 | 0 | 0.316 |
| *Eunotia microcephala* Krasske | 0 | 0 | 0 |  | 0.25 | 0 |
| *Eunotia minor* (Kutzing) Grunow | 0.8 | 1.08 | 0.65 | 0.316 | 0.25 | 0.633 |
| *Eunotia tenella* (Grunow) Hustedt | 0.2 | 0 | 0 | 0 | 0 | 0 |
| *Eunotia veneris* (Kutzing) De Toni | 0.2 | 0 | 0 | 0 | 0 | 0 |
| *Fragilaria capucina var. vaucheriae* (Kützing) Lange-Bertalot | 8.4 | 3.25 | 0 | 0.316 | 0.5 | 0 |
| *Fragilariforma virescens* Williams & Round | 1.2 | 0 | 0 | 0 | 0 | 0 |
| *Gomphonema acuminatum* Ehrenberg | 0.2 | 0 | 0 | 0.316 | 0 | 0 |
| *Gomphonema angustatum* (Kütz.) Rabenh | 0.8 | 0 | 0 | 0 | 0.25 | 0 |
| *Gomphonema insigne* W.Gregory | 0 | 0.65 | 0.65 | 0 | 0 | 0.316 |
| *Aulucoseira grunulutu* (Ehrenberg) | 0 | 0.43 | 0 | 0 | 0 | 0 |
| *Sellaphora americana* (Ehrenberg) D.G. Mann | 0 | 0 | 0 | 0.316 | 0.75 | 1.58 |
| *Craticula cuspidata* (Kutzing) D.G.Mann | 1.8 | 0.65 | 0.216 | 0 | 0 | 0.95 |
| *Navicula laevissima* Kützing *var. laevissima* | 0 | 0 | 0.216 | 0.316 | 0.25 | 0 |
| *Sellaphora pupula* (Kützing) Mereschkowsky | 0 | 0.65 | 0 | 0 | 0 | 0 |
| *Navicula radiosa* Kutzing | 0 | 0.216 | 0 | 0 | 0 | 0 |
| *Luticola cohnii* (Hilse) Mann var. cohnii | 0.2 | 0 | 0 | 0 | 0 | 0 |
| *Neidium ampliatum* (Ehrenberg) Krammer | 0 | 0.216 | 0 | 0 | 0 | 0 |
| *Nitzschia linearis* [(C. Agardh) W. Sm.](https://www.itis.gov/servlet/SingleRpt/RefRpt?search_type=author&search_id=author_id&search_id_value=161814) | 0.8 | 0 | 0 | 0 | 0 | 0 |
| *Nitzschia alpine* Hustedt | 0 | 0.43 | 0 | 0 | 0 | 0 |
| *Nitzschia sociabilis* Hustedt | 0.2 | 0.216 | 0 | 0 | 0 | 0 |
| *Pinnularia braunii* Grunow) Cleve | 1.8 | 0.216 | 0 | 0 | 0 | 0 |
| *Pinnularia major* (Kützing) Rabenhorst | 0.4 | 1.08 | 0 | 0 | 0 | 0 |
| *Pinnularia pulchra* Oestrup | 1 | 0.216 | 0 | 0 | 0 | 0 |
| *Surirella carpronii* Brébisson | 0.6 | 0 | 0 | 0 | 0 | 0 |
| *Iconella splendida* (Ehrenberg) Ruck & Nakov | 0 | 0.216 | 1.08 | 0.633 | 0.25 | 1.58 |
| *Fragilaria* *capucina* Desmazières | 0 | 0 | 0 | 0 | 0.25 | 0 |
| *Fragilaria ulna*(Nitzsch) Lange-Bertalot | 0 | 0.43 | 0.433 | 0.316 | 0 | 0 |
