# Supplementary material for: Benthic diatom communities and a comparative seasonal-based ecological quality assessment of a transboundary river in Bangladesh
Source: PLoS One. 2023 Oct 4;18(10):e0291751. doi: 10.1371/journal.pone.0291751 (PMC10550107; doi:10.1371/journal.pone.0291751)
Supplement: S2 Table — (DOCX) [file pone.0291751.s002.docx]

**Table S2a.The biovolume of benthic diatom (x10³ µm³/L) in the Sari-Goyain River, Bangladesh during the wet season.**

| Diatom | Sampling sites | | | | | | | | | |
| --- | --- | --- | --- | --- | --- | --- | --- | --- | --- | --- |
|  | WS1 | WS2 | WS3 | WS4 | WS5 | WS6 | WS7 | WS8 | WS9 | WS100 |
| *Amphora libyca* Ehrenberg | 0 | 0 | 0 | 0 | 0 | 0 | 0 | 55.24 | 0 | 0 |
| *Cymbella cursiformis* L. Hufford & Collins | 48.697 | 0 | 58.671 | 0 | 0 | 0 | 0 | 0 | 0 | 0 |
| *Encyonema turgidum* (Gregory) Grunow | 0 | 489.53 | 1174.87 | 1272.77 | 847.86 | 847.86 | 0 | 1435.3 | 0 | 293.71 |
| *Diploneis ovalis* (Hilse) Cleve | 0 | 0 | 0 | 0 | 0 | 0 | 0 | 107.77 | 0 | 0 |
| *Eunotia marina* Schrader | 0 | 0 | 58.82 | 0 | 0 | 0 | 0 | 0 | 0 | 0 |
| *Eunotia minor* (Kutzing) Grunow | 134.727 | 0 | 80.9176 | 0 | 0 | 0 | 0 | 0 | 0 | 606.688 |
| *Eunotia veneris* (Kutzing) De Toni | 0 | 0 | 0 | 0 | 0 | 0 | 0 | 193.76 | 0 | 0 |
| *Fragilaria capucina var. vaucheriae* (Kützing) Lange-Bertalot | 1826.39 | 0 | 3290.8 | 5923.44 | 1184.68 | 3565.03 | 822.7 | 2007.38 | 0 | 0 |
| *Gomphonema tergestinum* (Grunow) M. Schmidt | 0 | 0 | 27.02 | 0 | 0 | 0 | 0 | 0 | 0 | 0 |
| *Gomphonema angustatum*[(Kütz.) Rabenh.](https://www.itis.gov/servlet/SingleRpt/RefRpt?search_type=author&search_id=author_id&search_id_value=161690) | 0 | 0 | 0 | 0 | 0 | 0 | 0 | 646.33 | 0 | 176.27 |
| *Gomphonema incognitum* Reichardt, Jüttner & E.J.Cox | 0 | 0 | 29.68 | 0 | 0 | 0 | 0 | 0 | 0 | 0 |
| *Gomphonema insigne* W.Gregory | 287.52 | 1299.03 | 0 | 0 | 0 | 374.122 | 259.807 | 1586.55 | 0 | 259.807 |
| *Gomphonema longiceps* (Ehrenberg) | 0 | 0 | 0 | 0 | 187.45 | 124.87 | 0 | 0 | 0 | 0 |
| *Gomphonema olivaceoides*Hustedt | 0 | 0 | 16.52 | 0 | 0 | 0 | 0 | 0 | 0 | 0 |
| *Aulucoseira grunulutu* (Ehrenberg) | 0 | 0 | 0 | 0 | 540 | 0 | 0 | 0 | 0 | 0 |
| *Sellaphora americana* (Ehrenberg) D.G.Mann | 2798.9 | 0 | 0 | 0 | 0 | 1209.12 | 0 | 0 | 0 | 0 |
| *Craticula cuspidata* (Kutzing) D.G.Mann | 0 | 34.39 | 27.51 | 29.72 | 0 | 29.72 | 0 | 50.36 | 27.51 | 0 |
| *Luticolacohnii* (Hilse) Mann var. cohnii | 0 | 0 | 0 | 0 | 0 | 0 | 0 | 0 | 0 | 47.63 |
| *Neidium ampliatum* (Ehrenberg) Krammer | 0 | 0 | 0 | 0 | 0 | 0 | 1118.64 | 0 | 0 | 0 |
| *Pinnularia acrosphaeria* W.Smith | 0 | 0 | 0 | 0 | 955.58 | 0 | 0 | 0 | 0 | 0 |
| *Pinnularia braunii* (Grunow) Cleve | 0 | 0 | 975.5 | 175.59 | 0 | 0 | 0 | 447.1 | 325.16 | 0 |
| *Pinnularia hemiptera*  [(Kütz.) Rabenh.](https://www.itis.gov/servlet/SingleRpt/RefRpt?search_type=author&search_id=author_id&search_id_value=161690) |  | 0 | 0 | 214.81 | 0 | 0 | 0 | 0 | 0 | 0 |
| *Pinnularia major* (Kützing) Rabenhorst | 711.13 | 2141.95 | 856.783 | 0 | 0 | 1854.93 | 0 | 0 | 0 | 0 |
| *Pinnularia pulchra* Oestrup | 0 | 0 | 0 | 0 | 0 | 0 | 618.54 | 0 | 0 | 0 |
| *Pinnularia sp* | 0 | 0 | 1047.19 | 0 | 0 | 0 | 0 | 958.18 | 0 | 0 |
| *Stauroneis schroederi*Hustedt | 0 | 0 | 0 | 0 | 0 | 0 | 42.46 | 0 | 0 | 0 |
| *Strauroneis anceps* Ehrenberg | 0 | 22.55 | 0 | 0 | 0 | 0 | 0 | 0 | 0 | 0 |
| *Iconella splendida*(Ehrenberg) Ruck &Nakov | 0 | 0 | 0 | 0 | 0 | 0 | 0 | 169.99 | 0 | 0 |
| *Fragilaria ulna*(Nitzsch) Lange-Bertalot | 0 | 668.22 | 0 | 578.68 | 0 | 288.67 | 0 | 979.61 | 534.57 | 0 |

**Table S2b.The biovolume of benthic diatom (x10³** **µm³/L) in the Sari-Goyain River, Bangladesh during the dry season.**

| Diatom | Sampling sites | | | | | |
| --- | --- | --- | --- | --- | --- | --- |
|  | DS1 | DS2 | DS3 | DS4 | DS5 | DS6 |
| *Amphora libyca* Ehrenberg | 0 | 129.81 | 0 | 0 | 0 | 0 |
| *Encyonema turgidum* (Gregory) Grunow | 1174.87 | 1172.77 | 847.86 | 0 | 979.06 | 0 |
| *Diploneis ovalis* (Hilse) Cleve | 117.78 | 127.2 | 0 | 0 | 0 | 186.09 |
| *Eunotia microcephala* Krasske | 0 | 0 | 0 | 0 | 868.6 | 0 |
| *Eunotia minor* (Kutzing) Grunow | 323.67 | 436.95 | 262.98 | 127.84 | 101.14 | 256.1 |
| *Eunotia tenella* (Grunow) Hustedt | 1372.38 | 0 | 0 | 0 | 0 | 0 |
| *Eunotia veneris* (Kutzing) De Toni | 70.46 | 0 | 0 | 0 | 0 | 0 |
| *Fragilaria capucina var. vaucheriae* (Kützing) Lange-Bertalot | 46071.2 | 17825.17 | 0 | 1733.15 | 2742.33 | 0 |
| *Fragilariforma virescens* Williams & Round | 622.5 | 0 | 0 | 0 | 0 | 0 |
| *Gomphonema acuminatum* Ehrenberg | 0 | 0 | 0 | 214.37 | 0 | 0 |
| *Gomphonema angustatum* (Kütz.) Rabenh | 235.03 | 0 | 0 | 0 | 293.78 | 0 |
| *Gomphonema insigne* W.Gregory | 0 | 1125.83 | 1125.83 | 0 | 0 | 547.32 |
| *Aulucoseira grunulutu* (Ehrenberg) | 0 | 1075 | 0 | 0 | 0 | 0 |
| *Sellaphoraamericana* (Ehrenberg) D.G. Mann | 0 | 0 | 0 | 0 | 0 | 8844.54 |
| *Navicula cuspidate* (Kutzing) D.G. Mann | 247.67 | 89.43 | 29.72 | 0 | 0 | 130.71 |
| *Luticola cohnii* (Hilse) Mann var. cohnii | 63.51 | 0 | 0 | 0 | 0 | 0 |
| *Neidium ampliatum* (Ehrenberg) Krammer | 0 | 805.42 | 0 | 0 | 0 | 0 |
| *Nitzschia linearis* [(C. Agardh) W. Sm.](https://www.itis.gov/servlet/SingleRpt/RefRpt?search_type=author&search_id=author_id&search_id_value=161814) | 1468.69 | 0 | 0 | 0 | 0 | 0 |
| *Nitzschia sociabilis* Hustedt | 21.77 | 21.37 | 0 | 0 | 0 | 0 |
| *Pinnularia braunii* (Grunow) Cleve | 1463.25 | 175.59 | 0 | 0 | 0 | 0 |
| *Pinnularia major* (Kützing) Rabenhorst | 1713.56 | 4626.63 | 0 | 0 | 0 | 0 |
| *Pinnularia pulchra* Oestrup | 4123.64 | 890.7 | 0 | 0 | 0 | 0 |
| *Surirella carpronii* Brébisson | 51.27 | 0 | 0 | 0 | 0 | 0 |
| *Iconella splendida* (Ehrenberg) Ruck & Nakov | 0 | 200.65 | 1003.27 | 588.02 | 232.23 | 1467.75 |
| *Fragilaria* *capucina* Desmazières | 0 | 0 | 0 | 0 | 334.11 | 0 |
| *Fragilaria ulna*(Nitzsch) Lange-Bertalot | 0 | 574.67 | 578.68 | 422.31 | 0 | 0 |
