# Supplementary material for: Benthic diatom communities and a comparative seasonal-based ecological quality assessment of a transboundary river in Bangladesh
Source: PLoS One. 2023 Oct 4;18(10):e0291751. doi: 10.1371/journal.pone.0291751 (PMC10550107; doi:10.1371/journal.pone.0291751)
Supplement: S1 Appendix — (DOCX) [file pone.0291751.s003.docx]

**Benthic diatom communities and a comparative seasonal-based ecological quality assessment of a transboundary river Bangladesh**

Md Mehedi Hasan^1¶*^, Md Ataul Gani^1¶^, Md. Almujaddade Alfasane^2^. Mst. Ayesha ^2^, Khurshid Nahar^1^

**Appendices**

**Table A1. TDI of WS1 site in Sari-Goyain River, Bangladesh (*a* = abundance (proportion) of species in the sample, *v* = indicator value and s = pollution sensitivity of species)**

| Sl | Organisms | 1st | 2nd | 3rd | Abundance (×10^3^) ind/l | a | v | s | asv | av | WMS= $\sum asv$/$\sum av$ | TDI=(WMS*25)-25 |
| --- | --- | --- | --- | --- | --- | --- | --- | --- | --- | --- | --- | --- |
| 1 | *Cymbella cursiformis* L.Hufford & Collins | 1 | 0 | 0 | 0.166 | 0.33333 | 1 | 2 | 0.66666 | 0.33333 |  |  |
| 2 | *Eunotia minor*(Kützing) Grunow | 0 | 1 | 1 | 0.333 | 0.666667 | 3 | 1 | 2 | 2 |  |  |
| 3 | *Fragilaria capucina var. vaucheriae* (Kützing) Lange-Bertalot | 1 | 0 | 1 | 0.333 | 0.666667 | 1 | 2 | 1.333333 | 0.666667 |  |  |
| 4 | *Gomphonema insigne* W.Gregory | 1 | 0 | 0 | 0.166 | 0.333333 | 1 | 3 | 1 | 0.333333 |  |  |
| 5 | *Sellaphora americana* (Ehrenberg) D.G.Mann | 2 | 0 | 1 | 0.5 | 1 | 1 | 4 | 4 | 1 |  |  |
| 6 | *Pinnularia major* (Kützing) Rabenhorst | 1 | 0 | 0 | 0.166 | 0.333333 | 3 | 1 | 1 | 1 |  |  |
| 7 | *Fragilaria capucina*Desmazières | 0 | 1 | 0 | 0.166 | 0.333333 | 1 | 4 | 1.333333 | 0.333333 |  |  |
|  |  |  |  |  |  |  |  |  | 11.33333 | 5.666667 | 2 | 25 |

**Table A2. TDI of WS2 site in Sari-Goyain River, Bangladesh (*a* = abundance (proportion) of species in the sample, *v* = indicator value and s = pollution sensitivity of species)**

| Sl | Organisms | 1st | 2nd | 3rd | Abundance (×10^3^) ind/l | a | v | s | asv | av | WMS= $\sum asv$/$\sum av$ | TDI=(WMS*25)-25 |
| --- | --- | --- | --- | --- | --- | --- | --- | --- | --- | --- | --- | --- |
| 1 | *Encyonema elginense* (Krammer) D.G.Mann | 0 | 1 | 0 | 0.25 | 0.333333 | 1 | 2 | 0.666667 | 0.333333 |  |  |
| 2 | *Gomphonema insigne* W.Gregory | 0 | 1 | 2 | 0.75 | 1 | 1 | 3 | 3 | 1 |  |  |
| 3 | *Craticula cuspidata* (Kutzing) D.G.Mann | 0 | 0 | 1 | 0.25 | 0.333333 | 1 | 4 | 1.333333 | 0.333333 |  |  |
| 4 | *Pinnularia major* (Kützing) Rabenhorst | 0 | 1 | 1 | 0.5 | 0.666667 | 3 | 1 | 2 | 2 |  |  |
| 5 | *Strauroneis anceps* Ehrenberg | 1 | 0 | 0 | 0.25 | 0.333333 | 2 | 5 | 3.333333 | 0.666667 |  |  |
| 6 | *Fragilaria ulna*(Nitzsch) Lange-Bertalot | 2 | 0 | 0 | 0.5 | 0.666667 | 1 | 3 | 2 | 0.666667 |  |  |
|  |  |  |  |  |  |  |  |  | 12.33333 | 5 | 2.466666667 | 36.66666667 |

**Table A3. TDI of WS3 site in Sari-Goyain River, Bangladesh (*a* = abundance (proportion) of species in the sample, *v* = indicator value and s = pollution sensitivity of species)**

| Sl | Organisms | 1st | 2nd | 3rd | Abundance (×10^3^) ind/l | a | v | s | asv | av | WMS= $\sum asv$/$\sum av$ | TDI=(WMS*25)-25 |
| --- | --- | --- | --- | --- | --- | --- | --- | --- | --- | --- | --- | --- |
| 1 | *Cymbella cursiformis* L.Hufford & Collins | 0 | 0 | 1 | 0.2 | 0.333333 | 1 | 2 | 0.666667 | 0.333333 |  |  |
| 2 | *Encyonema elginense* (Krammer) D.G.Mann | 0 | 1 | 2 | 0.6 | 1 | 1 | 2 | 2 | 1 |  |  |
| 3 | *Eunotia marina* Schrader | 0 | 1 | 0 | 0.2 | 0.333333 | 3 | 1 | 1 | 1 |  |  |
| 4 | *Eunotia minor*(Kützing) Grunow | 1 | 0 | 0 | 0.2 | 0.333333 | 3 | 1 | 1 | 1 |  |  |
| 5 | *Fragilaria capucina var. vaucheriae* (Kützing) Lange-Bertalot | 1 | 2 | 0 | 0.6 | 1 | 1 | 2 | 2 | 1 |  |  |
| 6 | *Gomphomema tergestinum* (Grunow) M.Schmidt | 0 | 1 | 0 | 0.2 | 0.333333 | 1 | 3 | 1 | 0.333333 |  |  |
| 7 | *Gomphonema incognitum* Reichardt, Jüttner & E.J.Cox | 2 | 0 | 0 | 0.4 | 0.666667 | 1 | 3 | 2 | 0.666667 |  |  |
| 8 | *Gomphonema olivaceoides* Hustedt | 0 | 0 | 1 | 0.2 | 0.333333 | 3 | 2 | 2 | 1 |  |  |
| 9 | *Craticula cuspidata* (Kutzing) D.G.Mann | 0 | 0 | 1 | 0.2 | 0.333333 | 1 | 4 | 1.333333 | 0.333333 |  |  |
| 10 | *Pinnularia braunii* (Grunow) Cleve | 3 | 2 | 1 | 1.2 | 2 | 3 | 1 | 6 | 6 |  |  |
| 11 | *Pinnularia sp* | 0 | 0 | 1 | 0.2 | 0.333333 | 3 | 1 | 1 | 1 |  |  |
| 12 | *Fragilaria capucina var. amphicephala* (Kütz.) Lange-Bert. | 0 | 0 | 3 | 0.6 | 1 | 1 | 4 | 4 | 1 |  |  |
|  |  |  |  |  |  |  |  |  | 24 | 14.66667 | 1.636363636 | 15.90909091 |

**Table A4. TDI of WS4 site in Sari-Goyain River, Bangladesh (*a* = abundance (proportion) of species in the sample, *v* = indicator value and s = pollution sensitivity of species)**

| Sl | Organisms | 1st | 2nd | 3rd | Abundance (×10^3^) ind/l | a | v | s | asv | av | WMS= $\sum asv$/$\sum av$ | TDI=(WMS*25)-25 |
| --- | --- | --- | --- | --- | --- | --- | --- | --- | --- | --- | --- | --- |
| 1 | *Encyonema elginense* (Krammer) D.G.Mann | 1 | 2 | 0 | 0.65 | 1 | 1 | 2 | 2 | 1 |  |  |
| 2 | *Fragilaria capucina var. vaucheriae* (Kützing) Lange-Bertalot | 1 | 1 | 3 | 1.08 | 1.666667 | 1 | 2 | 3.333333 | 1.666667 |  |  |
| 3 | *Craticula cuspidata* (Kutzing) D.G.Mann | 0 | 0 | 1 | 0.216 | 0.333333 | 1 | 4 | 1.333333 | 0.333333 |  |  |
| 4 | *Pinnularia braunii* (Grunow) Cleve | 0 | 1 | 0 | 0.216 | 0.333333 | 3 | 1 | 1 | 1 |  |  |
| 5 | *Pinnularia hemiptera* (Kütz.) Rabenh | 0 | 1 | 0 | 0.216 | 0.333333 | 3 | 1 | 1 | 1 |  |  |
| 6 | *Fragilaria ulna*(Nitzsch) Lange-Bertalot | 1 | 0 | 1 | 0.433 | 0.666667 | 1 | 3 | 2 | 0.666667 |  |  |
|  |  |  |  |  |  |  |  |  | 10.66667 | 5.666667 | 1.882352941 | 22.05882353 |

**Table A5. TDI of WS5 site in Sari-Goyain River, Bangladesh (*a* = abundance (proportion) of species in the sample, *v* = indicator value and s = pollution sensitivity of species)**

| Sl | Organisms | 1st | 2nd | 3^rd^ | Abundance (×10^3^) ind/l | a | v | s | asv | av | WMS= $\sum asv$/$\sum av$ | TDI=(WMS*25)-25 |
| --- | --- | --- | --- | --- | --- | --- | --- | --- | --- | --- | --- | --- |
| 1 | *Encyonema elginense* (Krammer) D.G.Mann | 1 | 0 | 1 | 0.433 | 0.666667 | 1 | 2 | 1.333333 | 0.666667 |  |  |
| 2 | *Fragilaria capucina var. vaucheriae* (Kützing) Lange-Bertalot | 1 | 0 | 0 | 0.216 | 0.333333 | 1 | 2 | 0.666667 | 0.333333 |  |  |
| 3 | *Gomphonema longiceps* (Ehrenberg) | 0 | 3 | 0 | 0.65 | 1 | 1 | 3 | 3 | 1 |  |  |
| 4 | *Aulucoseira grunulutu* (Ehrenberg) | 1 | 0 | 0 | 0.216 | 0.333333 | 2 | 4 | 2.666667 | 0.666667 |  |  |
| 5 | *Pinnularia acrosphaeria* W.Smith | 0 | 2 | 0 | 0.433 | 0.666667 | 3 | 1 | 2 | 2 |  |  |
|  |  |  |  |  |  |  |  |  | 9.666667 | 4.666667 | 2.071428571 | 26.78571429 |

**Table A6. TDI of WS6 site in Sari-Goyain River, Bangladesh (*a* = abundance (proportion) of species in the sample, *v* = indicator value and s = pollution sensitivity of species)**

| Sl | | Organisms | 1st | 2nd | 3rd | Abundance (×10^3^) ind/l | a | v | s | asv | av | WMS= $\sum asv$/$\sum av$ | TDI=(WMS*25)-25 |
| --- | --- | --- | --- | --- | --- | --- | --- | --- | --- | --- | --- | --- | --- |
| 1 | *Encyonema elginense* (Krammer) D.G.Mann | | 2 | 0 | 0 | 0.433 | 0.666667 | 1 | 2 | 1.333333 | 0.666667 |  |  |
| 2 | *Eunotia minor*(Kützing) Grunow | | 0 | 0 | 1 | 0.216 | 0.333333 | 3 | 1 | 1 | 1 |  |  |
| 3 | *Fragilaria capucina var. vaucheriae* (Kützing) Lange-Bertalot | | 1 | 1 | 1 | 0.65 | 1 | 1 | 2 | 2 | 1 |  |  |
| 4 | *Gomphonema insigne* W.Gregory | | 0 | 1 | 0 | 0.216 | 0.333333 | 1 | 3 | 1 | 0.333333 |  |  |
| 5 | *Gomphonema longiceps* (Ehrenberg) | | 1 | 0 | 1 | 0.433 | 0.666667 | 1 | 3 | 2 | 0.666667 |  |  |
| 6 | *Sellaphora americana* (Ehrenberg) D.G.Mann | | 0 | 1 | 0 | 0.216 | 0.333333 | 1 | 4 | 1.333333 | 0.333333 |  |  |
| 7 | *Craticula cuspidata* (Kutzing) D.G.Mann | | 1 | 0 | 0 | 0.216 | 0.333333 | 1 | 4 | 1.333333 | 0.333333 |  |  |
| 8 | *Pinnularia major* (Kützing) Rabenhorst | | 0 | 0 | 2 | 0.433 | 0.666667 | 3 | 1 | 2 | 2 |  |  |
| 9 | *Fragilaria ulna*(Nitzsch) Lange-Bertalot | | 0 | 0 | 1 | 0.216 | 0.333333 | 1 | 3 | 1 | 0.333333 |  |  |
|  |  | |  |  |  |  |  |  |  | 13 | 6.666667 | 1.95 | 23.75 |

**Table A7. TDI of WS7 site in Sari-Goyain River, Bangladesh (*a* = abundance (proportion) of species in the sample, *v* = indicator value and s = pollution sensitivity of species)**

| Sl | Organisms | 1st | 2nd | 3rd | Abundance (×10^3^) ind/l | a | v | s | asv | av | WMS= $\sum asv$/$\sum av$ | TDI=(WMS*25)-25 |
| --- | --- | --- | --- | --- | --- | --- | --- | --- | --- | --- | --- | --- |
| 1 | *Fragilaria capucina var. vaucheriae* (Kützing) Lange-Bertalot | 0 | 1 | 0 | 0.15 | 0.333333 | 1 | 2 | 0.666667 | 0.333333 |  |  |
| 2 | *Gomphonema insigne* W.Gregory | 0 | 0 | 1 | 0.15 | 0.333333 | 1 | 3 | 1 | 0.333333 |  |  |
| 3 | *Neidium ampliatum* (Ehrenberg) Krammer | 1 | 1 | 0 | 0.3 | 0.666667 | 3 | 2 | 4 | 2 |  |  |
| 4 | *Pinnularia pulchra* Oestrup | 1 | 0 | 0 | 0.15 | 0.333333 | 3 | 1 | 1 | 1 |  |  |
| 5 | *Stauroneis schroederi* Hustedt | 1 | 0 | 0 | 0.15 | 0.333333 | 2 | 5 | 3.333333 | 0.666667 |  |  |
|  |  |  |  |  |  |  |  |  | 10 | 4.333333 | 2.307692308 | 32.69230769 |

**Table A8. TDI of WS8 site in Sari-Goyain River, Bangladesh (*a* = abundance (proportion) of species in the sample, *v* = indicator value and s = pollution sensitivity of species)**

| Sl | Organisms | 1st | 2nd | 3rd | Abundance (×10^3^) ind/l | a | v | s | asv | av | WMS= $\sum asv$/$\sum av$ | TDI=(WMS*25)-25 |
| --- | --- | --- | --- | --- | --- | --- | --- | --- | --- | --- | --- | --- |
| 1 | *Amphora libyca* (Ehrenberg) | 0 | 1 | 0 | 0.183 | 0.333333 | 1 | 5 | 1.666667 | 0.333333 |  |  |
| 2 | *Cymbella tumida* (Brébisson) Van Heurck | 0 | 1 | 0 | 0.183 | 0.333333 | 1 | 2 | 0.666667 | 0.333333 |  |  |
| 3 | *Encyonema elginense* (Krammer) D.G.Mann | 2 | 1 | 1 | 0.733 | 1.333333 | 1 | 2 | 2.666667 | 1.333333 |  |  |
| 4 | *Diploneis ovalis* (Hilse) Cleve | 0 | 0 | 1 | 0.183 | 0.333333 | 1 | 1 | 0.333333 | 0.333333 |  |  |
| 5 | *Eunotia veneris* (Kutzing) De Toni | 0 | 1 | 2 | 0.55 | 1 | 3 | 1 | 3 | 3 |  |  |
| 6 | *Fragilaria capucina var. vaucheriae* (Kützing) Lange-Bertalot | 0 | 0 | 2 | 0.366 | 0.666667 | 1 | 2 | 1.333333 | 0.666667 |  |  |
| 7 | *Gomphonema angustatum* (Kütz.) Rabenh | 3 | 0 | 0 | 0.55 | 1 | 2 | 1 | 2 | 2 |  |  |
| 8 | *Gomphonema insigne* W.Gregory | 3 | 1 | 1 | 0.916 | 1.666667 | 1 | 3 | 5 | 1.666667 |  |  |
| 9 | *Craticula cuspidata* (Kutzing) D.G. Mann | 0 | 1 | 1 | 0.366 | 0.666667 | 1 | 4 | 2.666667 | 0.666667 |  |  |
| 10 | *Sellaphora pupula* (Kützing) Mereschkowsky | 0 | 1 | 0 | 0.183 | 0.333333 | 1 | 4 | 1.333333 | 0.333333 |  |  |
| 11 | *Pinnularia braunii* (Grunow) Cleve | 1 | 1 | 1 | 0.55 | 1 | 3 | 1 | 3 | 3 |  |  |
| 12 | *Pinnularia sp* | 0 | 0 | 1 | 0.183 | 0.333333 | 3 | 1 | 1 | 1 |  |  |
| 13 | *Iconella splendida* (Ehrenberg) Ruck & Nakov | 0 | 1 | 0 | 0.183 | 0.333333 | 1 | 3 | 1 | 0.333333 |  |  |
| 14 | *Fragilaria ulna*(Nitzsch) Lange-Bertalot | 1 | 1 | 2 | 0.733 | 1.333333 | 1 | 3 | 4 | 1.333333 |  |  |
|  |  |  |  |  |  |  |  |  | 29.66667 | 16.33333 | 1.816326531 | 20.40816327 |

**Table A9. TDI of WS9 site in Shari-Goyain River, Bangladesh (*a* = abundance (proportion) of species in the sample, *v* = indicator value and s = pollution sensitivity of species)**

| Sl | Organisms | 1st | 2nd | 3rd | Abundance (×10^3^) ind/l | a | v | s | asv | av | WMS= $\sum asv$/$\sum av$ | TDI=(WMS*25)-25 |
| --- | --- | --- | --- | --- | --- | --- | --- | --- | --- | --- | --- | --- |
| 1 | *Craticula cuspidata* (Kutzing) D.G. Mann | 0 | 0 | 1 | 0.2 | 0.333333 | 1 | 4 | 1.333333 | 0.333333 |  |  |
| 2 | *Pinnularia braunii* (Grunow) Cleve | 1 | 1 | 0 | 0.4 | 0.666667 | 3 | 1 | 2 | 2 |  |  |
| 3 | *Fragilaria ulna*(Nitzsch) Lange-Bertalot | 1 | 1 | 0 | 0.4 | 0.666667 | 1 | 3 | 2 | 0.666667 |  |  |
|  |  |  |  |  |  |  |  |  | 5.333333 | 3 | 1.777777778 | 19.44444444 |

**Table A10. TDI of WS10 site in Shari-Goyain River, Bangladesh (*a* = abundance (proportion) of species in the sample, *v* = indicator value and s = pollution sensitivity of species)**

| Sl | Organisms | 1st | 2nd | 3rd | Abundance (×10^3^) ind/l | a | v | s | asv | av | WMS= $\sum asv$/$\sum av$ | TDI=(WMS*25)-25 |
| --- | --- | --- | --- | --- | --- | --- | --- | --- | --- | --- | --- | --- |
| 1 | *Encyonema elginense* (Krammer) D.G. Mann | 0 | 0 | 1 | 0.15 | 0.333333 | 1 | 2 | 0.666667 | 0.333333 |  |  |
| 2 | *Eunotia minor*(Kützing) Grunow | 0 | 0 | 1 | 0.15 | 0.333333 | 3 | 1 | 1 | 1 |  |  |
| 3 | *Gomphonema angustatum* (Kütz.) Rabenh | 0 | 1 | 0 | 0.15 | 0.333333 | 2 | 1 | 0.666667 | 0.666667 |  |  |
| 4 | *Gomphonema insigne* W.Gregory | 0 | 1 | 0 | 0.15 | 0.333333 | 1 | 3 | 1 | 0.333333 |  |  |
| 5 | *Luticola cohnii* (Hilse) Mann var. cohnii | 0 | 1 | 0 | 0.15 | 0.333333 | 1 | 4 | 1.333333 | 0.333333 |  |  |
|  |  |  |  |  |  |  |  |  | 4.666667 | 2.666667 | 1.75 | 18.75 |

**Table A11. TDI of DS1site in Shari-Goyain River, Bangladesh (*a* = abundance (proportion) of species in the sample, *v* = indicator value and s = pollution sensitivity of species)**

| Sl | Organisms | 1st | 2nd | 3rd | Abundance (×10^3^) ind/l | | a | v | s | asv | av | WMS= $\sum asv$/$\sum av$ | TDI=(WMS*25)-25 |
| --- | --- | --- | --- | --- | --- | --- | --- | --- | --- | --- | --- | --- | --- |
| 1 | *Encyonema elginense* (Krammer) D.G. Mann | 0 | 1 | 2 | 0.6 | | 1 | 1 | 2 | 2 | 1 |  |  |
| 2 | *Diploneis ovalis* (Hilse) Cleve | 0 | 1 | 0 | 0.2 | | 0.333333 | 1 | 1 | 0.333333 | 0.333333 |  |  |
| 3 | *Eunotia minor*(Kützing) Grunow | 3 | 0 | 1 | 0.8 | | 1.333333 | 3 | 1 | 4 | 4 |  |  |
| 4 | *Eunotia tenella* | 0 | 0 | 1 | 0.2 | | 0.333333 | 3 | 1 | 1 | 1 |  |  |
| 5 | *Eunotia veneris* (Kutzing) De Toni | 1 | 0 | 0 | 0.2 | | 0.333333 | 3 | 1 | 1 | 1 |  |  |
| 6 | *Fragilaria capucina var. vaucheriae* (Kützing) Lange-Bertalot | 12 | 15 | 15 | 8.4 | | 14 | 1 | 2 | 28 | 14 |  |  |
| 7 | *Fragilariforma virescens* Williams & Round | 1 | 4 | 1 | 1.2 | | 2 | 1 | 2 | 4 | 2 |  |  |
| 8 | *Gomphonema angustatum* (Kütz.) Rabenh | 0 | 0 | 1 | 0.2 | | 0.333333 | 2 | 1 | 0.666667 | 0.666667 |  |  |
| 9 | *Sellaphora americana* (Ehrenberg) D.G. Mann | 2 | 1 | 1 | 0.8 | | 1.333333 | 1 | 4 | 5.333333 | 1.333333 |  |  |
| 10 | *Craticula cuspidata* (Kutzing) D.G. Mann | 3 | 3 | 3 | 1.8 | | 3 | 1 | 4 | 12 | 3 |  |  |
| 11 | *Navicula grammei* | 0 | 1 | 0 | 0.2 | | 0.333333 | 1 | 4 | 1.333333 | 0.333333 |  |  |
| 12 | *Navicula laevissima* Kützing *var. laevissima* | 1 | 0 | 0 | 0.2 | | 0.333333 | 1 | 4 | 1.333333 | 0.333333 |  |  |
| 13 | *Luticola cohnii* (Hilse) Mann var. cohnii | 1 | 0 | 0 | 0.2 | | 0.333333 | 1 | 4 | 1.333333 | 0.333333 |  |  |
| 14 | *Sellaphora pupula* (Kützing) Mereschkowsky | 1 | 0 | 0 | 0.2 | | 0.333333 | 1 | 4 | 1.333333 | 0.333333 |  |  |
| 15 | Navicula sp | 0 | 2 | 0 | 0.4 | | 0.666667 | 1 | 4 | 2.666667 | 0.666667 |  |  |
| 16 | *Nitzschia linearis* [(C. Agardh) W. Sm.](https://www.itis.gov/servlet/SingleRpt/RefRpt?search_type=author&search_id=author_id&search_id_value=161814) | 1 | 2 | 1 | 0.8 | | 1.333333 | 1 | 4 | 5.333333 | 1.333333 |  |  |
| 17 | *Nitzschia sociabilis* Hustedt | 1 | 0 | 0 | 0.2 |  | 0.333333 | 1 | 4 | 1.333333 | 0.333333 |  |  |
| 18 | *Pinnularia braunii*  (Grunow) Cleve | 3 | 3 | 3 | 1.8 |  | 3 | 3 | 1 | 9 | 9 |  |  |
| 19 | *Pinnularia major* (Kützing) Rabenhorst | 0 | 0 | 2 | 0.4 |  | 0.666667 | 3 | 1 | 2 | 2 |  |  |
| 20 | *Pinnularia pulchra* Oestrup | 4 | 1 | 0 | 1 |  | 1.666667 | 3 | 1 | 5 | 5 |  |  |
| 21 | *Surirella carpronii* Brébisson | 1 | 0 | 2 | 0.6 |  | 1 | 1 | 3 | 3 | 1 |  |  |
|  |  |  |  |  |  |  |  |  |  | 92 | 49 | 1.87755102 | 21.93877551 |

**Table A12. TDI of DS2 site in Shari-Goyain River, Bangladesh (*a* = abundance (proportion) of species in the sample, *v* = indicator value and s = pollution sensitivity of species)**

| Sl | Organisms | 1st | 2nd | 3rd | Abundance (×10^3^) ind/l | a | v | s | asv | av | WMS= $\sum asv$/$\sum av$ | TDI=(WMS*25)-25 |
| --- | --- | --- | --- | --- | --- | --- | --- | --- | --- | --- | --- | --- |
| 1 | *Amphora libyca* (Ehrenberg) | 1 | 1 | 0 | 0.43 | 0.666667 | 1 | 5 | 3.333333 | 0.666667 |  |  |
| 2 | *Cymbella tumida* (Brébisson) Van Heurck | 0 | 1 | 0 | 0.216 | 0.333333 | 1 | 2 | 0.666667 | 0.333333 |  |  |
| 3 | *Encyonema elginense* (Krammer) D.G. Mann | 0 | 4 | 0 | 0.866 | 1.333333 | 1 | 2 | 2.666667 | 1.333333 |  |  |
| 4 | *Encyonema elginense* (Krammer) D.G. Mann | 2 | 0 | 1 | 0.65 | 1 | 1 | 2 | 2 | 1 |  |  |
| 5 | *Diploneis ovalis* (Hilse) Cleve | 0 | 0 | 1 | 0.216 | 0.333333 | 1 | 1 | 0.333333 | 0.333333 |  |  |
| 6 | *Eunotia minor*(Kützing) Grunow | 1 | 1 | 3 | 1.08 | 1.666667 | 3 | 1 | 5 | 5 |  |  |
| 7 | *Fragilaria capucina var. vaucheriae* (Kützing) Lange-Bertalot | 5 | 2 | 8 | 3.25 | 5 | 1 | 2 | 10 | 5 |  |  |
| 8 | *Gomphonema insigne* W.Gregory | 0 | 3 | 0 | 0.65 | 1 | 1 | 3 | 3 | 1 |  |  |
| 9 | *Aulacoseira granulata* (Ehrenberg) Simonsen | 0 | 0 | 2 | 0.43 | 0.666667 | 2 | 4 | 5.333333 | 1.333333 |  |  |
| 10 | *Sellaphora pupula* (Kützing) Mereschkowsky | 2 | 1 | 0 | 0.65 | 1 | 1 | 4 | 4 | 1 |  |  |
| 11 | *Navicula radiosa* Kutzing | 1 | 0 | 0 | 0.216 | 0.333333 | 1 | 4 | 1.333333 | 0.333333 |  |  |
| 12 | Navicula sp | 1 | 0 | 0 | 0.216 | 0.333333 | 1 | 4 | 1.333333 | 0.333333 |  |  |
| 13 | *Craticula cuspidata* (Kutzing) D.G.Mann | 1 | 1 | 1 | 0.65 | 1 | 1 | 4 | 4 | 1 |  |  |
| 14 | *Neidium ampliatum* (Ehrenberg) Krammer | 0 | 1 | 0 | 0.216 | 0.333333 | 3 | 2 | 2 | 1 |  |  |
| 15 | *Nitzschia alpina* Hustedt | 0 | 2 | 0 | 0.43 | 0.666667 | 1 | 4 | 2.666667 | 0.666667 |  |  |
| 16 | *Nitzschia sociabilis* Hustedt | 0 | 1 | 0 | 0.216 | 0.333333 | 1 | 4 | 1.333333 | 0.333333 |  |  |
| 17 | *Pinnularia braunii* (Grunow) Cleve | 1 | 0 | 0 | 0.216 | 0.333333 | 3 | 1 | 1 | 1 |  |  |
| 18 | Pinnularia laevissima | 1 | 0 | 0 | 0.216 | 0.333333 | 3 | 1 | 1 | 1 |  |  |
| 19 | *Pinnularia major* (Kützing) Rabenhorst | 4 | 1 | 0 | 1.08 | 1.666667 | 3 | 1 | 5 | 5 |  |  |
| 20 | *Pinnularia pulchra* Oestrup | 0 | 0 | 1 | 0.216 | 0.333333 | 3 | 1 | 1 | 1 |  |  |
| 21 | *Iconella splendida* (Ehrenberg) Ruck & Nakov | 0 | 0 | 1 | 0.216 | 0.333333 | 1 | 3 | 1 | 0.333333 |  |  |
| 22 | *Fragilaria ulna*(Nitzsch) Lange-Bertalot | 0 | 0 | 2 | 0.43 | 0.666667 | 1 | 3 | 2 | 0.666667 |  |  |
|  |  |  |  |  |  |  |  |  | 60 | 29.66667 | 2.02247191 | 25.56179775 |

**Table A13. TDI of DS3 site in Sari-Goyain River, Bangladesh (*a* = abundance (proportion) of species in the sample, *v* = indicator value and s = pollution sensitivity of species)**

| Sl | Organisms | 1st | 2nd | 3rd | Abundance (×10^3^) ind/l | a | v | s | asv | av | WMS= $\sum asv$/$\sum av$ | TDI=(WMS*25)-25 |
| --- | --- | --- | --- | --- | --- | --- | --- | --- | --- | --- | --- | --- |
| 1 | *Encyonema elginense* (Krammer) D.G. Mann | 2 | 0 | 0 | 0.433 | 0.666667 | 1 | 2 | 1.333333 | 0.666667 |  |  |
| 2 | *Eunotia minor*(Kützing) Grunow | 0 | 1 | 2 | 0.65 | 1 | 3 | 1 | 3 | 3 |  |  |
| 3 | *Gomphonema insigne* W.Gregory | 0 | 0 | 3 | 0.65 | 1 | 1 | 3 | 3 | 1 |  |  |
| 4 | *Gomphonema sp* | 1 | 0 | 0 | 0.216 | 0.333333 | 1 | 3 | 1 | 0.333333 |  |  |
| 5 | *Craticula cuspidata* (Kutzing) D.G. Mann | 0 | 1 | 0 | 0.216 | 0.333333 | 1 | 4 | 1.333333 | 0.333333 |  |  |
| 6 | *Navicula laevissima* Kützing *var. laevissima* | 0 | 1 | 0 | 0.216 | 0.333333 | 1 | 4 | 1.333333 | 0.333333 |  |  |
| 7 | *Iconella splendida* (Ehrenberg) Ruck & Nakov | 0 | 3 | 2 | 1.08 | 1.666667 | 1 | 3 | 5 | 1.666667 |  |  |
| 8 | *Fragilaria ulna*(Nitzsch) Lange-Bertalot | 0 | 2 | 0 | 0.433 | 0.666667 | 1 | 3 | 2 | 0.666667 |  |  |
|  |  |  |  |  |  |  |  |  | 18 | 8 | 2.25 | 31.25 |

**Table A14. TDI of DS4 site in Sari-Goyain River, Bangladesh (*a* = abundance (proportion) of species in the sample, *v* = indicator value and s = pollution sensitivity of species)**

| Station 04, DRY SEASON | |  |  |  |  |  |  |  |  |  |  |  |  |
| --- | --- | --- | --- | --- | --- | --- | --- | --- | --- | --- | --- | --- | --- |
| Sl | Organisms | 1st | 2nd | 3rd | Abundance (×10^3^) ind/l |  | a | v | s | asv | av | WMS= $\sum asv$/$\sum av$ | TDI=(WMS*25)-25 |
| 1 | *Eunotia minor*(Kützing) Grunow | 0 | 1 | 0 | 0.316 |  | 0.333333 | 3 | 1 | 1 | 1 |  |  |
| 2 | *Fragilaria capucina var. vaucheriae* (Kützing) Lange-Bertalot | 0 | 1 | 0 | 0.316 |  | 0.333333 | 1 | 2 | 0.666667 | 0.333333 |  |  |
| 3 | *Gomphonema acuminatum*  Ehrenberg | 0 | 1 | 0 | 0.316 |  | 0.333333 | 1 | 3 | 1 | 0.333333 |  |  |
| 4 | *Sellaphora americana* (Ehrenberg) D.G. Mann | 0 | 0 | 1 | 0.316 |  | 0.333333 | 1 | 4 | 1.333333 | 0.333333 |  |  |
| 5 | *Navicula laevissima* Kützing *var. laevissima* | 0 | 0 | 1 | 0.316 |  | 0.333333 | 1 | 4 | 1.333333 | 0.333333 |  |  |
| 6 | *Iconella splendida* (Ehrenberg) Ruck & Nakov | 1 | 1 | 0 | 0.633 |  | 0.666667 | 1 | 3 | 2 | 0.666667 |  |  |
| 7 | *Fragilaria ulna*(Nitzsch) Lange-Bertalot | 1 | 0 | 0 | 0.316 |  | 0.333333 | 1 | 3 | 1 | 0.333333 |  |  |
|  |  |  |  |  |  |  |  |  |  | 8.333333 | 3.333333 | 2.5 | 37.5 |

**Table A15. TDI of DS5 site in Sari-Goyain River, Bangladesh (*a* = abundance (proportion) of species in the sample, *v* = indicator value and s = pollution sensitivity of species)**

| Sl | Organisms | 1st | 2nd | 3rd | Abundance (×10^3^) ind/l | a | v | s | asv | av | WMS= $\sum asv$/$\sum av$ | TDI=(WMS*25)-25 |
| --- | --- | --- | --- | --- | --- | --- | --- | --- | --- | --- | --- | --- |
| 1 | *Encyonema elginense* (Krammer) D.G. Mann | 1 | 0 | 1 | 0.5 | 0.666667 | 1 | 2 | 1.333333 | 0.666667 |  |  |
| 2 | *Eunotia microcephala* Krasske | 0 | 0 | 1 | 0.25 | 0.333333 | 3 | 1 | 1 | 1 |  |  |
| 3 | *Eunotia minor*(Kützing) Grunow | 1 | 0 | 0 | 0.25 | 0.333333 | 3 | 1 | 1 | 1 |  |  |
| 4 | *Fragilaria capucina var. vaucheriae* (Kützing) Lange-Bertalot | 0 | 1 | 1 | 0.5 | 0.666667 | 1 | 2 | 1.333333 | 0.666667 |  |  |
| 5 | *Gomphonema angustatum* (Kütz.) Rabenh | 1 | 0 | 0 | 0.25 | 0.333333 | 1 | 3 | 1 | 0.333333 |  |  |
| 6 | *Sellaphora americana* (Ehrenberg) D.G. Mann | 0 | 1 | 2 | 0.75 | 1 | 1 | 4 | 4 | 1 |  |  |
| 7 | *Navicula laevissima* Kützing *var. laevissima* | 0 | 0 | 1 | 0.25 | 0.333333 | 1 | 4 | 1.333333 | 0.333333 |  |  |
| 8 | *Iconella splendida* (Ehrenberg) Ruck & Nakov | 1 | 0 | 0 | 0.25 | 0.333333 | 1 | 3 | 1 | 0.333333 |  |  |
| 9 | *Fragilaria* *capucina* Desmazières | 0 | 1 | 0 | 0.25 | 0.333333 | 1 | 3 | 1 | 0.333333 |  |  |
|  |  |  |  |  |  |  |  |  | 13 | 5.666667 | 2.294117647 | 32.35294118 |

**Table A16. TDI of DS6 site in Sari-Goyain River, Bangladesh (*a* = abundance (proportion) of species in the sample, *v* = indicator value and s = pollution sensitivity of species)**

| Sl | Organisms | 1st | 2nd | 3rd | Abundance (×10^3^) ind/l | a | v | s | asv | av | WMS= $\sum asv$/$\sum av$ | TDI=(WMS*25)-25 |
| --- | --- | --- | --- | --- | --- | --- | --- | --- | --- | --- | --- | --- |
| 1 | *Diploneis ovalis* (Hilse) Cleve | 0 | 1 |  | 0.316 | 0.5 | 1 | 1 | 0.5 | 0.5 |  |  |
| 2 | *Eunotia minor*(Kützing) Grunow | 1 | 0 | 1 | 0.633 | 0.666667 | 3 | 1 | 2 | 2 |  |  |
| 3 | *Gomphonema angustum* (Kütz.) Rabenh | 0 | 0 | 1 | 0.316 | 0.333333 | 1 | 3 | 1 | 0.333333 |  |  |
| 4 | *Gomphonema insigne* W.Gregory | 0 | 0 | 1 | 0.316 | 0.333333 | 1 | 3 | 1 | 0.333333 |  |  |
| 5 | *Sellaphora americana* (Ehrenberg) D.G. Mann | 1 | 2 | 2 | 1.58 | 1.666667 | 1 | 4 | 6.666667 | 1.666667 |  |  |
| 6 | *Craticula cuspidata* (Kutzing) D.G.Mann | 1 | 0 | 2 | 0.95 | 1 | 1 | 4 | 4 | 1 |  |  |
| 7 | *Iconella splendida* (Ehrenberg) Ruck & Nakov | 3 | 2 | 0 | 1.58 | 1.666667 | 1 | 3 | 5 | 1.666667 |  |  |
|  |  |  |  |  |  |  |  |  | 20.16667 | 7.5 | 2.688888889 | 42.22222222 |

**Table A17. WQI of WS1 site in Sari-Goyain river, Bangladesh**

| Parameters4 | Standard Permissible Value (Vs) by ECR 1997 | Monitored value, (Vn) | Quality Rating,  Q_i_=100(Vn -Vi)/(Vs-Vi) | Relative Weight (W_i_)= 1/Vs | W_i_ * Q_i_ |
| --- | --- | --- | --- | --- | --- |
| pH | 8.5 | 7.2 | 13.33333 | 0.117647 | 1.568627 |
| DO (mg/l) | 6 | 13.4 | 13.95349 | 0.166667 | 2.325581 |
| EC (µS/cm) | 2500 | 84.5 | 3.38 | 0.0004 | 0.001352 |
| TDS (mg/L) | 1000 | 55.3 | 5.53 | 0.001 | 0.00553 |
| PO_4_^3-^ (mg/L) | 6 | 0 | 0 | 0.166667 | 0 |
| NO_3_^-^ (mg/L) | 10 | 0.43 | 4.3 | 0.1 | 0.43 |
|  |  |  | ∑ Qi=40.49682 | ∑ Wi =  0.55238 | ∑Wi * Qi=  4.331091 |
| $WQI=\sum_{i=1}^{n} \frac{\mathrm{WiQi}}{\sum_{i=1}^{n} \mathrm{Wi}}$= 7.84077586 (Excellent) | | | | | |

**Table A18. WQI of WS2 site in Shari-Goyain River, Bangladesh**

| Parameters | Standard Permissible Value (Vs) by ECR 1997 | Monitored value, (Vn) | Quality Rating,Q_i_=100(Vn -Vi)/(Vs-Vi) | Relative Weight (W_i_)= 1/Vs | W_i_ * Q_i_ |
| --- | --- | --- | --- | --- | --- |
| pH | 8.5 | 7.3 | 20 | 0.117647 | 2.352941 |
| DO (mg/l) | 6 | 13.2 | 16.27907 | 0.166667 | 2.713178 |
| EC (µS/cm) | 2500 | 90 | 3.6 | 0.0004 | 0.00144 |
| TDS (mg/L) | 1000 | 57.7 | 5.77 | 0.001 | 0.00577 |
| PO_4_^3-^ (mg/L) | 6 | 0 | 0 | 0.166667 | 0 |
| NO_3_^-^ (mg/L) | 10 | 0.43 | 4.3 | 0.1 | 0.43 |
|  |  |  | ∑ Qi=  49.94907 | ∑ Wi =0.55238 | ∑Wi * Qi=5.503329 |
| $WQI=\sum_{i=1}^{n} \frac{\mathrm{WiQi}}{\sum_{i=1}^{n} \mathrm{Wi}}$= 9.96293415 (Excellent) | | | | | |

**Table A19. WQI of WS3 site in Sari-Goyain River, Bangladesh**

| Parameters | Standard Permissible Value (Vs) by ECR 1997 | Monitored value, (Vn) | Quality Rating,Q_i_=100(Vn -Vi)/(Vs-Vi) | Relative Weight (W_i_)= 1/Vs | W_i_ * Q_i_ |
| --- | --- | --- | --- | --- | --- |
| pH | 8.5 | 7.4 | 26.66667 | 0.117647 | 3.137255 |
| DO (mg/l) | 6 | 13 | 18.60465 | 0.166667 | 3.100775 |
| EC (µS/cm) | 2500 | 65.8 | 2.632 | 0.0004 | 0.001053 |
| TDS (mg/L) | 1000 | 42.5 | 4.25 | 0.001 | 0.00425 |
| PO_4_^3-^ (mg/L) | 6 | 0 | 0 | 0.166667 | 0 |
| NO_3_^-^ (mg/L) | 10 | 0.23 | 2.3 | 0.1 | 0.23 |
|  |  |  | ∑ Qi=  54.45332 | ∑ Wi =  0.55238 | ∑Wi * Qi=  6.473333 |
| $WQI=\sum_{i=1}^{n} \frac{\mathrm{WiQi}}{\sum_{i=1}^{n} \mathrm{Wi}}$= 11.7189766 (Excellent) | | | | | |

**Table A20. WQI of WS4 site in Sari-Goyain River, Bangladesh**

| Parameters | Standard Permissible Value (Vs) by ECR 1997 | Monitored value, (Vn) | Quality Rating,Q_i_=100(Vn -Vi)/(Vs-Vi) | Relative Weight (W_i_)= 1/Vs | W_i_ * Q_i_ |
| --- | --- | --- | --- | --- | --- |
| pH | 6.5 | 6.8 | 40 | 0.153846 | 6.153846 |
| DO (mg/l) | 6 | 12.4 | 25.5814 | 0.166667 | 4.263566 |
| EC (µS/cm) | 2500 | 65.5 | 2.62 | 0.0004 | 0.001048 |
| TDS (mg/L) | 1000 | 42.5 | 4.25 | 0.001 | 0.00425 |
| PO_4_^3-^ (mg/L) | 6 | 0 | 0 | 0.166667 | 0 |
| NO_3_^-^ (mg/L) | 10 | 0.26 | 2.6 | 0.1 | 0.26 |
|  |  |  | ∑ Qi=  75.0514 | ∑ Wi =  0.588579 | ∑Wi * Qi=  10.68271 |
| $WQI=\sum_{i=1}^{n} \frac{\mathrm{WiQi}}{\sum_{i=1}^{n} \mathrm{Wi}}$= 18.149987 (Excellent) | | | | | |

**Table A21 WQI of WS5 site in Sari-Goyain River, Bangladesh**

| Parameters | Standard Permissible Value (Vs) by ECR 1997 | Monitored value, (Vn) | Quality Rating,Q_i_=100(Vn -Vi)/(Vs-Vi) | Relative Weight (W_i_)= 1/Vs | W_i_ * Q_i_ |
| --- | --- | --- | --- | --- | --- |
| pH | 8.5 | 7.1 | 6.666667 | 0.117647 | 0.784314 |
| DO (mg/l) | 6 | 13.4 | 13.95349 | 0.166667 | 2.325581 |
| EC (µS/cm) | 2500 | 68.4 | 2.736 | 0.0004 | 0.001094 |
| TDS (mg/L) | 1000 | 44.1 | 4.41 | 0.001 | 0.00441 |
| PO_4_^3-^ (mg/L) | 6 | 0.00186 | 0.031 | 0.166667 | 0.005167 |
| NO_3_^-^ (mg/L) | 10 | 0.34 | 3.4 | 0.1 | 0.34 |
|  |  |  | ∑ Qi=  31.19716 | ∑ Wi =  0.55238 | ∑Wi * Qi=  3.460566 |
| $WQI=\sum_{i=1}^{n} \frac{\mathrm{WiQi}}{\sum_{i=1}^{n} \mathrm{Wi}}$= 6.26482445 (Excellent) | | | | | |

**Table A22. WQI of WS6 site in Sari-Goyain River, Bangladesh**

| Parameters | Standard Permissible Value (Vs) by ECR 1997 | Monitored value, (Vn) | Quality Rating,Q_i_=100(Vn -Vi)/(Vs-Vi) | Relative Weight (W_i_)= 1/Vs | W_i_ * Q_i_ |
| --- | --- | --- | --- | --- | --- |
| pH | 8.5 | 7 | 0 | 0.117647 | 0 |
| DO (mg/l) | 6 | 13.5 | 12.7907 | 0.166667 | 2.131783 |
| EC (µS/cm) | 2500 | 69.2 | 2.768 | 0.0004 | 0.001107 |
| TDS (mg/L) | 1000 | 45.5 | 4.55 | 0.001 | 0.00455 |
| PO_4_^3-^ (mg/L) | 6 | 0.01346 | 0.224333 | 0.166667 | 0.037389 |
| NO_3_^-^ (mg/L) | 10 | 0.26 | 2.6 | 0.1 | 0.26 |
|  |  |  | ∑ Qi=  22.93303 | ∑ Wi =  0.55238 | ∑Wi * Qi=  2.434829 |
| $WQI=\sum_{i=1}^{n} \frac{\mathrm{WiQi}}{\sum_{i=1}^{n} \mathrm{Wi}}$= 4.40788462 (Excellent) | | | | | |

**Table A23. WQI of WS7 site in Sari-Goyain River, Bangladesh**

| Parameters | Standard Permissible Value (Vs) by ECR 1997 | Monitored value, (Vn) | Quality Rating,Q_i_=100(Vn -Vi)/(Vs-Vi) | Relative Weight (W_i_)= 1/Vs | W_i_ * Q_i_ |
| --- | --- | --- | --- | --- | --- |
| pH | 6.5 | 6.8 | 40 | 0.153846 | 6.153846 |
| DO (mg/l) | 6 | 12.6 | 23.25581 | 0.166667 | 3.875969 |
| EC (µS/cm) | 2500 | 34.4 | 1.376 | 0.0004 | 0.00055 |
| TDS (mg/L) | 1000 | 21.6 | 2.16 | 0.001 | 0.00216 |
| PO_4_^3-^ (mg/L) | 6 | 0.00331 | 0.055167 | 0.166667 | 0.009194 |
| NO_3_^-^ (mg/L) | 10 | 0.32 | 3.2 | 0.1 | 0.32 |
|  |  |  | ∑ Qi=  70.04698 | ∑ Wi =  0.588579 | ∑Wi * Qi=  10.36172 |
| $WQI=\sum_{i=1}^{n} \frac{\mathrm{WiQi}}{\sum_{i=1}^{n} \mathrm{Wi}}$= 17.604623 (Excellent) | | | | | |

**Table A24. WQI of WS8 site in Sari-Goyain River, Bangladesh**

| Parameters | Standard Permissible Value (Vs) by ECR 1997 | Monitored value, (Vn) | Quality Rating,Q_i_=100(Vn -Vi)/(Vs-Vi) | Relative Weight (W_i_)= 1/Vs | W_i_ * Q_i_ |
| --- | --- | --- | --- | --- | --- |
| pH | 8.5 | 7.4 | 26.66667 | 0.117647 | 3.137255 |
| DO (mg/l) | 6 | 13 | 18.60465 | 0.166667 | 3.100775 |
| EC (µS/cm) | 2500 | 55 | 2.2 | 0.0004 | 0.00088 |
| TDS (mg/L) | 1000 | 37.2 | 3.72 | 0.001 | 0.00372 |
| PO_4_^3-^ (mg/L) | 6 | 0.00186 | 0.031 | 0.166667 | 0.005167 |
| NO_3_^-^ (mg/L) | 10 | 0.32 | 3.2 | 0.1 | 0.32 |
|  |  |  | ∑ Qi=  54.42232 | ∑ Wi =  0.55238 | ∑Wi * Qi=  6.567797 |
| $WQI=\sum_{i=1}^{n} \frac{\mathrm{WiQi}}{\sum_{i=1}^{n} \mathrm{Wi}}$= 11.889989 (Excellent) | | | | | |

**Table A25. WQI of WS9 site in Sari-Goyain River, Bangladesh**

| Parameters | Standard Permissible Value (Vs) by ECR 1997 | Monitored value, (Vn) | Quality Rating,Q_i_=100(Vn -Vi)/(Vs-Vi) | Relative Weight (W_i_)= 1/Vs | W_i_ * Q_i_ |
| --- | --- | --- | --- | --- | --- |
| pH | 8.5 | 7 | 0 | 0.117647 | 0 |
| DO (mg/l) | 6 | 13.6 | 11.62791 | 0.166667 | 1.937984 |
| EC (µS/cm) | 2500 | 57.4 | 2.296 | 0.0004 | 0.000918 |
| TDS (mg/L) | 1000 | 37.3 | 3.73 | 0.001 | 0.00373 |
| PO_4_^3-^ (mg/L) | 6 | 0.00114 | 0.019 | 0.166667 | 0.003167 |
| NO_3_^-^ (mg/L) | 10 | 0.23 | 2.3 | 0.1 | 0.23 |
|  |  |  | ∑ Qi=  19.97291 | ∑ Wi =  0.55238 | ∑Wi * Qi=  2.1758 |
| $WQI=\sum_{i=1}^{n} \frac{\mathrm{WiQi}}{\sum_{i=1}^{n} \mathrm{Wi}}$= 3.93895148 (Excellent) | | | | | |

**Table A26. WQI of WS10 site in Sari-Goyain River, Bangladesh**

| Parameters | Standard Permissible Value (Vs) by ECR 1997 | Monitored value, (Vn) | Quality Rating,Q_i_=100(Vn -Vi)/(Vs-Vi) | Relative Weight (W_i_)= 1/Vs | W_i_ * Q_i_ |
| --- | --- | --- | --- | --- | --- |
| pH | 8.5 | 7.2 | 13.33333 | 0.117647 | 1.568627 |
| DO (mg/l) | 6 | 13.3 | 15.11628 | 0.166667 | 2.51938 |
| EC (µS/cm) | 2500 | 46 | 1.84 | 0.0004 | 0.000736 |
| TDS (mg/L) | 1000 | 9.1 | 0.91 | 0.001 | 0.00091 |
| PO_4_^3-^ (mg/L) | 6 | 0.01636 | 0.272667 | 0.166667 | 0.045444 |
| NO_3_^-^ (mg/L) | 10 | 0.23 | 2.3 | 0.1 | 0.23 |
|  |  |  | ∑ Qi=  33.77228 | ∑ Wi =  0.55238 | ∑Wi * Qi=  4.365098 |
| $WQI=\sum_{i=1}^{n} \frac{\mathrm{WiQi}}{\sum_{i=1}^{n} \mathrm{Wi}}$= 7.90234013 (Excellent) | | | | | |

**Table A27. WQI of DS1 in Sari-Goyain River, Bangladesh**

| Parameters | Standard Permissible Value (Vs) by ECR 1997 | Monitored value, (Vn) | Quality Rating,Q_i_=100(Vn -Vi)/(Vs-Vi) | Relative Weight (W_i_)= 1/Vs | W_i_ * Q_i_ |
| --- | --- | --- | --- | --- | --- |
| pH | 8.5 | 7.7 | 46.66667 | 0.117647 | 5.490196 |
| DO (mg/l) | 6 | 12.7 | 22.09302 | 0.166667 | 3.682171 |
| EC (µS/cm) | 2500 | 89.5 | 3.58 | 0.0004 | 0.001432 |
| TDS (mg/L) | 1000 | 68.9 | 6.89 | 0.001 | 0.00689 |
| PO_4_^3-^ (mg/L) | 6 | 0 | 0 | 0.166667 | 0 |
| NO_3_^-^ (mg/L) | 10 | 0.205 | 2.05 | 0.1 | 0.205 |
|  |  |  | ∑ Qi=  81.27969 | 0.55238 | ∑Wi * Qi=  9.385689 |
| $WQI=\sum_{i=1}^{n} \frac{\mathrm{WiQi}}{\sum_{i=1}^{n} \mathrm{Wi}}$= 16.9913501 (Excellent) | | | | | |

**Table A28. WQI of DS1 site in Sari-Goyain River, Bangladesh**

| Parameters | Standard Permissible Value (Vs) by ECR 1997 | Monitored value, (Vn) | Quality Rating,Q_i_=100(Vn -Vi)/(Vs-Vi) | Relative Weight (W_i_)= 1/Vs | W_i_ * Q_i_ |
| --- | --- | --- | --- | --- | --- |
| pH | 8.5 | 7.3 | 20 | 0.117647 | 2.352941 |
| DO (mg/l) | 6 | 13.5 | 12.7907 | 0.166667 | 2.131783 |
| EC (µS/cm) | 2500 | 99.2 | 3.968 | 0.0004 | 0.001587 |
| TDS (mg/L) | 1000 | 65.3 | 6.53 | 0.001 | 0.00653 |
| PO_4_^3-^ (mg/L) | 6 | 0.00068 | 0.011333 | 0.166667 | 0.001889 |
| NO_3_^-^ (mg/L) | 10 | 0.24 | 2.4 | 0.1 | 0.24 |
|  |  |  | ∑ Qi=  45.70003 | ∑ Wi =  0.55238 | ∑Wi * Qi=  4.73473 |
| $WQI=\sum_{i=1}^{n} \frac{\mathrm{WiQi}}{\sum_{i=1}^{n} \mathrm{Wi}}$= 8.57150304 (Excellent) | | | | | |

**Table A29. WQI of DS3 site in Sari-Goyain River, Bangladesh**

| Parameters | Standard Permissible Value (Vs) by ECR 1997 | Monitored value, (Vn) | Quality Rating,Q_i_=100(Vn -Vi)/(Vs-Vi) | Relative Weight (W_i_)= 1/Vs | W_i_ * Q_i_ |
| --- | --- | --- | --- | --- | --- |
| pH | 8.5 | 8.9 | 126.6667 | 0.117647 | 14.90196 |
| DO (mg/l) | 6 | 11 | 41.86047 | 0.166667 | 6.976744 |
| EC (µS/cm) | 2500 | 85.6 | 3.424 | 0.0004 | 0.00137 |
| TDS (mg/L) | 1000 | 65.8 | 6.58 | 0.001 | 0.00658 |
| PO_4_^3-^ (mg/L) | 6 | 0.0556 | 0.926667 | 0.166667 | 0.154444 |
| NO_3_^-^ (mg/L) | 10 | 0.3 | 3 | 0.1 | 0.3 |
|  |  |  | ∑ Qi=  182.4578 | ∑ Wi =  0.55238 | ∑Wi * Qi=  22.3411 |
| $WQI=\sum_{i=1}^{n} \frac{\mathrm{WiQi}}{\sum_{i=1}^{n} \mathrm{Wi}}$= 40.4451341 (Good) | | | | | |

**Table A30. WQI of DS4 site in Sari-Goyain River, Bangladesh**

| Parameters | Standard Permissible Value (Vs) by ECR 1997 | Monitored value, (Vn) | Quality Rating,Q_i_=100(Vn -Vi)/(Vs-Vi) | Relative Weight (W_i_)= 1/Vs | W_i_ * Q_i_ |
| --- | --- | --- | --- | --- | --- |
| pH | 8.5 | 8.8 | 120 | 0.117647 | 14.11765 |
| DO (mg/l) | 6 | 11 | 41.86047 | 0.166667 | 6.976744 |
| EC (µS/cm) | 2500 | 86.9 | 3.476 | 0.0004 | 0.00139 |
| TDS (mg/L) | 1000 | 64.5 | 6.45 | 0.001 | 0.00645 |
| PO_4_^3-^ (mg/L) | 6 | 0 | 0 | 0.166667 | 0 |
| NO_3_^-^ (mg/L) | 10 | 0.304 | 3.04 | 0.1 | 0.304 |
|  |  |  | ∑ Qi=  174.8265 | ∑ Wi =  0.55238 | ∑Wi * Qi=  21.40623 |
| $WQI=\sum_{i=1}^{n} \frac{\mathrm{WiQi}}{\sum_{i=1}^{n} \mathrm{Wi}}$= 38.7527 (Good) | | | | | |

**Table A31. WQI of DS5 site in Sari-Goyain River, Bangladesh**

| Parameters | Standard Permissible Value (Vs) by ECR 1997 | Monitored value, (Vn) | Quality Rating,Q_i_=100(Vn -Vi)/(Vs-Vi) | Relative Weight (W_i_)= 1/Vs | W_i_ * Q_i_ |
| --- | --- | --- | --- | --- | --- |
| pH | 8.5 | 8.9 | 126.6667 | 0.117647 | 14.90196 |
| DO (mg/l) | 6 | 11 | 41.86047 | 0.166667 | 6.976744 |
| EC (µS/cm) | 2500 | 86.6 | 3.464 | 0.0004 | 0.001386 |
| TDS (mg/L) | 1000 | 63.5 | 6.35 | 0.001 | 0.00635 |
| PO_4_^3-^ (mg/L) | 6 | 0 | 0 | 0.166667 | 0 |
| NO_3_^-^ (mg/L) | 10 | 0.241 | 2.41 | 0.1 | 0.241 |
|  |  |  | ∑ Qi=  180.7511 | ∑ Wi =  0.55238 | ∑Wi * Qi=  22.12744 |
| $WQI=\sum_{i=1}^{n} \frac{\mathrm{WiQi}}{\sum_{i=1}^{n} \mathrm{Wi}}$= 40.0583382 (Good) | | | | | |

**Table A32. WQI of DS6 site in Sari-Goyain River, Bangladesh**

| Parameters | Standard Permissible Value (Vs) by ECR 1997 | Monitored value, (Vn) | Quality Rating,Q_i_=100(Vn -Vi)/(Vs-Vi) | Relative Weight (W_i_)= 1/Vs | W_i_ * Q_i_ |
| --- | --- | --- | --- | --- | --- |
| pH | 8.5 | 8.9 | 126.6667 | 0.117647 | 14.90196 |
| DO (mg/l) | 6 | 11 | 41.86047 | 0.166667 | 6.976744 |
| EC (µS/cm) | 2500 | 83.6 | 3.344 | 0.0004 | 0.001338 |
| TDS (mg/L) | 1000 | 63.1 | 6.31 | 0.001 | 0.00631 |
| PO_4_^3-^ (mg/L) | 6 | 0 | 0 | 0.166667 | 0 |
| NO_3_^-^ (mg/L) | 10 | 0.26 | 2.6 | 0.1 | 0.26 |
|  |  |  | ∑ Qi=  180.7811 | ∑ Wi =  0.55238 | ∑Wi * Qi=  22.14635 |
| $WQI=\sum_{i=1}^{n} \frac{\mathrm{WiQi}}{\sum_{i=1}^{n} \mathrm{Wi}}$= 40.0925755 (Good) | | | | | |
